# Functionality Versus Sustainability for PLA in MEX 3D Printing: The Impact of Generic Process Control Factors on Flexural Response and Energy Efficiency

**DOI:** 10.3390/polym15051232

**Published:** 2023-02-28

**Authors:** Markos Petousis, Nectarios Vidakis, Nikolaos Mountakis, Emmanuel Karapidakis, Amalia Moutsopoulou

**Affiliations:** 1 Department of Mechanical Engineering, Hellenic Mediterranean University, 71410 Heraklion, Greece; 2 Electrical and Computer Engineering Department, Hellenic Mediterranean University, 71410 Heraklion, Greece

**Keywords:** Polylactic Acid (PLA), optimization, Material Extrusion (MEX), energy consumption, energy efficiency, flexural strength, Taguchi analysis, Robust Design, Fused Filament Fabrication (FFF)

## Abstract

Process sustainability vs. mechanical strength is a strong market-driven claim in Material Extrusion (MEX) Additive Manufacturing (AM). Especially for the most popular polymer, Polylactic Acid (PLA), the concurrent achievement of these opposing goals may become a puzzle, especially since MEX 3D-printing offers a variety of process parameters. Herein, multi-objective optimization of material deployment, 3D printing flexural response, and energy consumption in MEX AM with PLA is introduced. To evaluate the impact of the most important generic and device-independent control parameters on these responses, the Robust Design theory was employed. Raster Deposition Angle (RDA), Layer Thickness (LT), Infill Density (ID), Nozzle Temperature (NT), Bed Temperature (BT), and Printing Speed (PS) were selected to compile a five-level orthogonal array. A total of 25 experimental runs with five specimen replicas each accumulated 135 experiments. Analysis of variances and reduced quadratic regression models (RQRM) were used to decompose the impact of each parameter on the responses. The ID, RDA, and LT were ranked first in impact on printing time, material weight, flexural strength, and energy consumption, respectively. The RQRM predictive models were experimentally validated and hold significant technological merit, for the proper adjustment of process control parameters per the MEX 3D-printing case.

## 1. Introduction

Manufacturing processes are among the critical parameters affecting sustainability [1] and their consequences on the environment [2,3]. Therefore, adopting sustainable methods is an evolving trend [4]. Sustainability can be evaluated by the level of the measures it is influenced by, such as energy consumption [5,6], with the manufacturing sector consuming 42% of energy worldwide [7]. AM is among the processes with a strong increase in its use as a manufacturing method, due to its productivity, among other aspects [8]. AM characteristics constitute the process as one with increased sustainability features, such as the use of recycled materials as raw material materials [9,10] and minimized waste [11,12]. Still, regarding the energy consumption of the AM process, although studies have been presented [13,14], there is no clear outcome, especially compared to the traditional manufacturing processes. Additional research is required, focusing on the performance and the parameters of each AM process, for example, binder jetting [15], MEX [16], etc.

In the MEX 3D printing method, research is extensive regarding the performance of parts made by polymers, such as PLA [17,18,19], ABS [20,21], PA12 [22,23,24,25], TPU [26,27], and PC [28,29,30,31], among others. The effect of the MEX properties on the mechanical performance of pure polymers [17,18,21,22,26,28] and in composites form [23,32] has been investigated and reported. Most of the research is experimental and for the analysis of the results, statistical and modeling tools, such as the full factorial Design of Experiments [28], the Taguchi design [17,18,21,25,26,27], regression analysis [22], and neural networks [23], have been employed. Regarding energy consumption though, the literature is rather limited [33,34].

The most widely used polymer in MEX 3D printing is PLA [34], attributed to several aspects of its performance, such as its mechanical properties, its processability [35], its biocompatibility [36,37,38,39], and its eco-friendliness, as it comes from natural amyl sources [35,36]. PLA is widely used in packaging and culinary applications [40]. Due to its biocompatibility, it has been used in medical applications, for implants [41], orthopedics [42], and scaffolds [36,37,38,39]. PLA, due to its eco-friendliness, positively contributes to the sustainability of the applications in which it is used. As expected, due to its popularity, PLA has been thoroughly studied in MEX 3D printing for its behavior and performance in the pure form [43] and as a matrix material in polymers [44,45,46,47,48]. For the analysis of the experimental findings, modeling tools have been applied (Taguchi design, artificial neural networks, etc.) [38,49,50,51,52,53,54,55,56,57,58]. The quality characteristics (dimensional accuracy, porosity, and surface roughness) of the MEX 3D printed parts with the PLA polymer have also been reported, with the experimental results processed with such types of modeling tools [18]. Research on the PLA polymer has also been extended in Hybrid Additive Manufacturing applications, combining the MEX AM process with friction stir welding for applications requiring parts with dimensions beyond the MEX 3D printers’ capabilities [59], and CO_2_ laser cutting to achieve better characteristics on the 3D printed parts surface quality [60].

Although research is extensive on the PLA polymer in MEX 3D printing, the required energy, contributing to the sustainability aspect of the material, for the fabrication of parts with the process has not been reported (and as a result has not been optimized) yet. Research has shown that the energy demands for the fabrication of parts with the 3D printing process are influenced by the settings used [34]. Additionally, the selected 3D printing settings for the fabrication of parts with the MEX process highly impact the performance of the parts [17,22].

The study herein tries to find the optimum set of 3D printing settings for the production of parts with the Fused Filament Fabrication (FFF) MEX 3D printing process made with the PLA polymer, with which the energy demands are minimized and simultaneously, the parts exhibit maximized mechanical properties under flexural loading. The response of the parts under flexural loading was selected to be investigated, as this is a common type of loading in mechanical parts, and both tensile and compression stresses are developed in the parts during this type of loading. An experimental approach was used in the work. Specimens were 3D printed and tested in the flexural test following the ASTM D790-02 international standard. Six (with three levels each) generic, machine-independent 3D printing settings were chosen to be simultaneously evaluated, forming a Taguchi L25 orthogonal array. Five replicas were manufactured and tested for each experimental run (set of 3D printing settings). The statistical analysis results were further processed with regression analysis. The reliability of the analysis was calculated and prediction models as functions of the six 3D printing settings were compiled. The accuracy of these prediction models was evaluated with two confirmation runs and it was found to be more than sufficient for the prediction of the metrics studied. Overall, it was found that by using specific 3D printing setting values, the energy consumption for the manufacturing of PLA parts with the FFF MEX 3D printing process, can be significantly reduced. At the same time, the flexural strength is highly affected by the 3D printing setting values used during the manufacture of the parts. Differences reaching 300% were found in the flexural strength of the parts, by using different values (among the levels studied herein) in the 3D printing settings. No set of parameter values optimized both the energy consumption and the flexural strength. Still, it was found that it is possible to build parts with better mechanical properties, using modest energy resources. The complex effects of the control parameters and their levels in the metrics assessed were highlighted verifying the need for the conducted analysis.

## 2. Materials and Methods

### 2.1. Experimental Process and Samples Characterization

**Figure 1:**
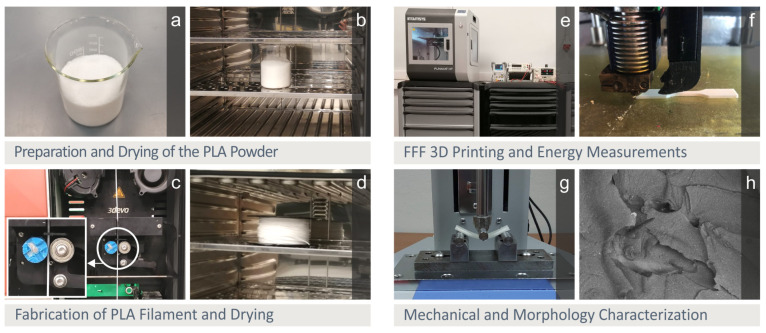
Experimental process (**a**) PLA raw material, (**b**) drying process for the raw material, (**c**) extrusion process for filament production, (**d**) drying of the filament, (**e**) measurements for the energy consumption during the FFF MEX 3D printing process, (**f**) FFF MEX 3D printing, (**g**) tensile experiments, (**h**) SEM images acquisition for the morphological characteristics’ evaluation.

In Figure 1, screenshots from the different steps followed herein are presented, depicting the implemented experimental procedure. PLA in fine powder form (Figure 1a, type 3052D, Plastika Kritis S.A., Heraklion, Greece) was dried in a laboratory oven (Figure 1b, 60 °C for 24 h) before being extruded to FFF MEX 3D printing 1.75 mm in diameter filament (Figure 1c, 3devo precision, Utrecht, the Netherlands, fan 80 %, 3.5 rpm, heat zone temperatures used no 1 170 °C, no 2 190 °C, no 3 185 °C, no 4 170 °C). The produced filament was also dried (Figure 1d, 60 °C for 4 h) to ensure no humidity is trapped in the polymer and used as the raw for the FFF MEX 3D printing process which was implemented employing a Funmat HT 3D printer (Figure 1e,f, Intamsys, Shanghai, China).

To ensure that the temperatures used in the two extrusion processes that the PLA polymer undergoes with the experimental methodology followed (filament extrusion, nozzle extrusion during 3D printing) do not affect its stability or cause any degradation that would have an impact on the experimental results, its thermal properties were examined. To achieve that, TGA (Perkin Elmer Diamond TG/TDA, Waltham, MA, USA, 40–550 °C, 10 °C/min, nitrogen purge gas) and DSC (Perkin Elmer Diamond DSC, Waltham, MA, USA, 50–300 °C, 10 °C/min, air atmosphere) measurements were taken.

**Figure 2:**
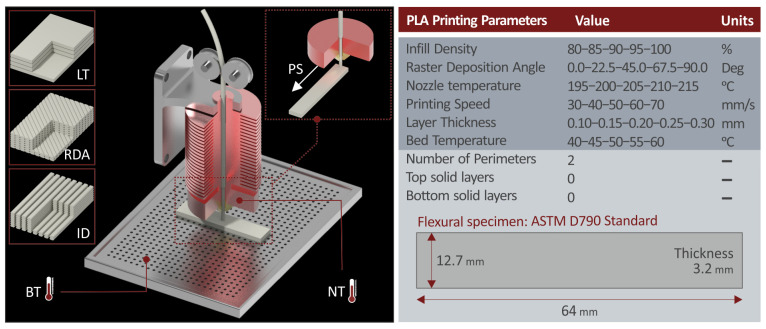
Three-dimensional printing with the FFF MEX process: 3D printing settings visual representation, their levels are depicted on the right side of the figure. The geometry of the specimens manufactured with the FFF MEX 3D printing process following the ASTM D790-02 standard.

The 3D printing settings are visually presented to be more comprehensive in Figure 2. Their values were determined from preliminary experiments and the existing literature [17]. They are depicted in Figure 2. as well, along with the geometry of the flexural test specimens built, following the corresponding international standard, i.e., ASTM D790-02. For each set of 3D printing parameters (experimental runs), five specimens were fabricated with the FFF MEX 3D printing process.

During the FFF MEX 3D printing process, the energy consumed by the Funmat HT 3D printer to produce the flexural test specimens was monitored (Figure 1e, Rigol DM3058E, RIGOL Technologies, Shanghai, China). The stopwatch method was applied [61]. After the flexural tests (Figure 1g, Imada-MX2 tester, Imada Inc., Illinois, United States, three-point-bending, 52 mm span, 10 mm/min elongation speed, ASTM D790-02), the morphological characteristics of the samples were evaluated with SEM (JEOL JSM 6362LV, Jeol Ltd., Peabody, MA, USA, high-vacuum mode, 20 kV, Au sputtered samples) and an optical stereoscope (Figure 1h, Kern OKO 1, 5MP ODC 832 camera, KERN, Balingen, Germany).

### 2.2. Energy Metrics

The energy consumption can be distinguished into three main phases: (i) machine startup, (ii) 3D printing process, and (iii) machine shutdown and can be calculated utilizing the equations below [34]:

(1) $$E_{total}=E_{thermal}+E_{motion}+E_{auxiliary}$$

where:

(2) $$E_{thermal}=E_{heating}+E_{cooling}$$

$E_{motion}$ is the consumed energy by the motors of the 3D printer and

(3) $$E_{auxiliary}=E_{startup}+E_{steadystate}+E_{shutdown}$$

of the electronics and the remaining parts of the 3D printer.

The Specific Printing Energy index is calculated:

(4) $$SPE=\frac{EPC}{w}\;\left[\mathrm{MJ}/g\right]$$

The Specific Printing Power index is calculated:

(5) $$SPP=\frac{EPC}{PT\cdot w}\cdot 10^{3}\;\left[\mathrm{kW}/g\right]$$

where Energy Printing Consumption (*EPC*) represents the energy used by the 3D printer ($E_{total}$), *w* the actual weight of each specimen, and *PT* the actual printing time for each experimental run.

### 2.3. Taguchi Design, Statistical and Regression Analysis

Herein, six generic, machine-independent 3D printing settings were simultaneously studied for their effect on the energy consumption and flexural properties of FFF MEX 3D printed parts. The interactions between them and the importance of each parameter and each level on the metrics were investigated. The 3D printing settings studies were Raster Deposition Angle (RDA, deg), Layer Thickness (LT, mm), Infill Density (ID, %), Nozzle Temperature (NT, °C), Bed Temperature (BT, °C), and Printing Speed (PS, mm/min). Each parameter was studied with three different levels and five replicas were tested in each case. Both the parameters and their levels were identified in accordance with the corresponding literature [17]. Such an approach on a full factorial experimental scheme would require 5 × 3^6^ experiments, which is not possible to be implemented within the context of the study. Therefore, the Taguchi design approach was followed [62]. An L25 orthogonal array was compiled.

A statistical analysis of the experimental results followed. Eight indicators were evaluated against the control parameters (3D printing settings), i.e., FFF MEX 3D printing time—s, 3D printed specimens’ weight—g, flexural strength—MPa, the flexural modulus of elasticity—MPa, flexural toughness—MJ/m^3^, EPC—MJ, SPE—MJ/g, and SPP—KW/g. These indicators provide insight into both the mechanical properties of the PLA parts under flexural loading and the required energy for their construction with the FFF MEX 3D printing process with the different 3D printing setting values (and as a result the effect of each value in the aforementioned indicators). A regression analysis was afterward conducted to evaluate the reliability of the results and the formation of prediction models for future use in real application environments. These prediction models calculate the aforementioned indicators as a function of the six 3D printing settings studied. To further evaluate their accuracy, two additional experiments were conducted (confirmation runs), using the 3D printing settings that produced the optimum results for flexural strength and EPC respectively in each experiment. Five specimens were also tested in each one of these two additional experiments.

## 3. Results

### 3.1. PLA Polymer Thermal Behavior and Morphological Characteristics of the 3D Printed Samples

**Figure 3:**
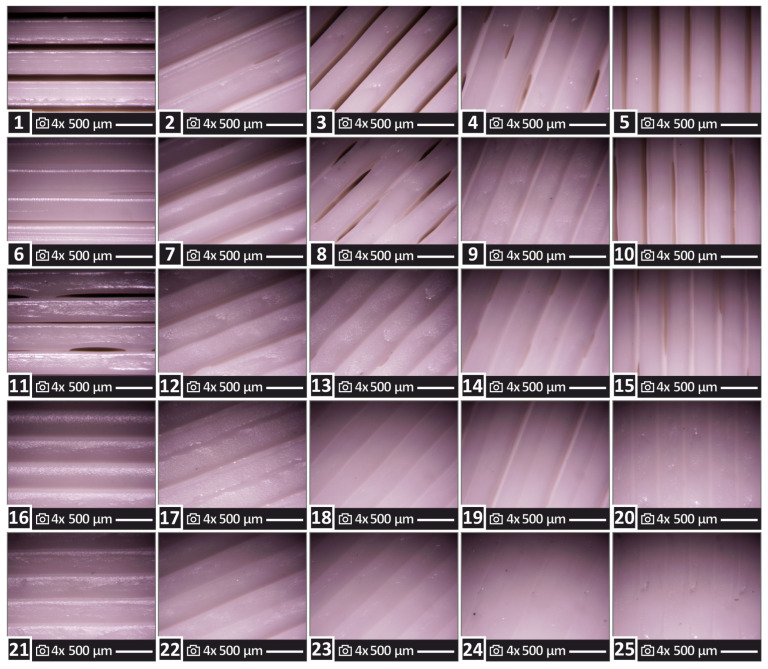
Optical stereoscope images are 4× magnification depicting the top surface on one of the five samples evaluated for each one of the twenty-five runs. The 3D printing structure differences among the samples can be evaluated in the images. In each image, the number corresponds to the run of the sample shown.

Figure 3 shows optical stereoscope images at 4× magnification of the top surface of a sample for each one of the twenty-five runs. A randomly selected sample out of the five tested in each run is shown in each image. A good quality 3D printing structure without voids or defects is observable in all runs. This indicates that the parameters in both the filament extrusion and the FFF MEX 3D printing process were suitable for the PLA polymer. Still, due to the differences in the 3D printing settings between the runs, the structure of the samples differs, as expected, and the differences are conceivable in the images.

**Figure 4:**
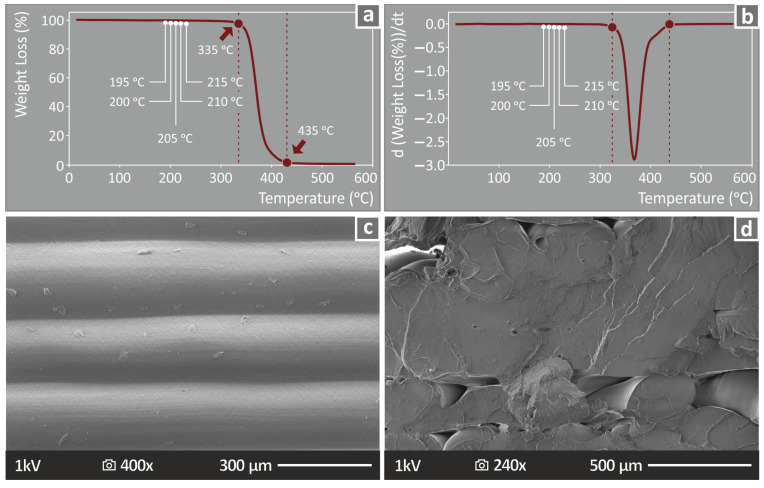
(**a**) Weight loss (%) vs. temperature (°C) graph produced on the TGA measurements of the specific PLA grade studied herein, (**b**) corresponding d(weight loss)/dt graph produced during the DSC measurements, and SEM images from (**c**) the side surface, (**d**) the fracture surface of a randomly selected sample, after the experimental testing with flexural loading.

Figure 4a shows the thermal degradation curve (weight loss vs. temperature) of the specific PLA polymer studied, as it was derived from the TGA. The corresponding DSC curve is shown in Figure 4b. Both the produced curves indicate that the temperatures used in the extrusion processes (filament production and 3D printing) do not have any impact on the thermal properties of the PLA polymer, thus they do not influence in any way the experimental findings of the study.

The morphology of the samples was further examined with SEM. Figure 4c shows the side surface of a sample, in which a perfect fusion between the layers is observed. The shape of the layers is also rather uniform. Slight deviations from the linear shape and in the thickness of the layers can be observed. Regarding the fracture area in Figure 4d, filament tearing is not observed [63]. Strands failed in a rather brittle failure with minimum deformation observed. Pores and voids are visible in the fracture area. This is the expected structure for FFF MEX 3D-printed polymeric materials [64].

### 3.2. Taguchi Design and Experimental Results

The Taguchi design of experiment with the six 3D printing settings (control parameters) and their levels (L25 orthogonal array) are cited in Table 1. The experimental results (mean values and deviations from the five experiments per run) for the eight in total (energy and flexural properties) metrics studied in the twenty-five runs performed are cited in Table 2 and Table 3. The analytic results for each experiment repetition are presented in the Supplementary Data provided with the study.

**Figure 5:**
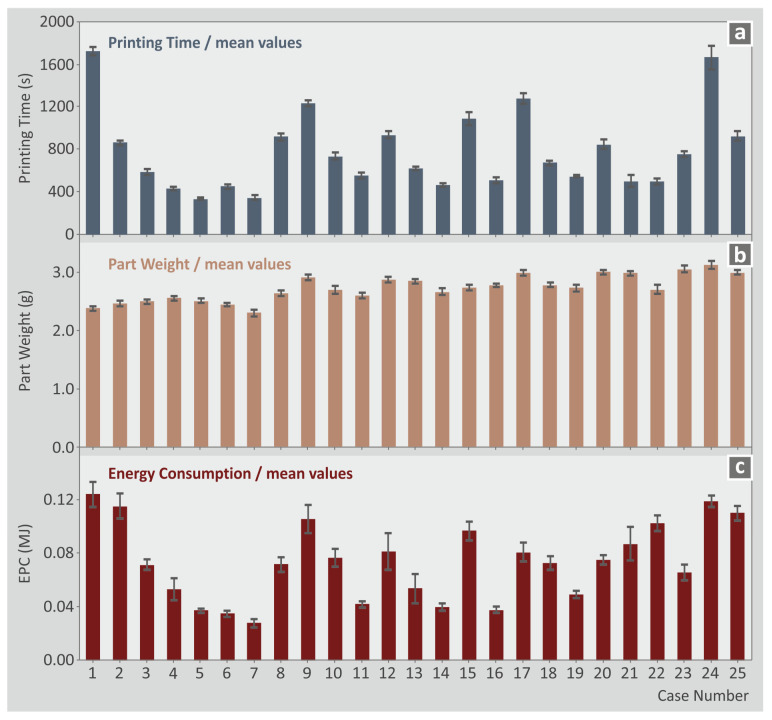
Experimental results mean values and deviation for three metrics (for the twenty-five runs) (**a**) printing time (s), (**b**) part weight (g), (**c**) EPC (MJ).

The mean values and the variation for the printing time, part weight, and EPC for the twenty-five runs are shown in a bar chart in Figure 5. This type of layout highlights the changes among the cases assessed for these metrics in a more vivid manner, adding to the thoroughness of the differences between the experimental findings and the significance of the 3D printing settings in the functionality of the manufactured parts. Such differences in the response metrics values justify the need for further analysis of the results. A strong correlation between the printing time and the EPC is observed, as a similar pattern in the bars for the two metrics is observed. The part weight does not seem to follow the same trend, showing a different pattern in the response values of the metric. Overall, the part weight is not significantly changing throughout the runs. In all response metric values, the calculated deviation is rather small, showing the reliability of the experimental results.

### 3.3. Statistical Analysis

**Figure 6:**
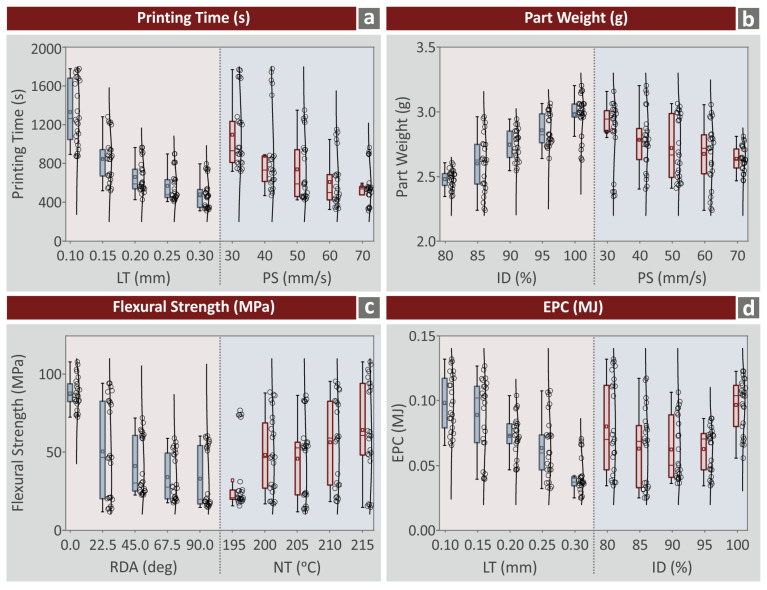
Box plot diagrams for four of the metrics evaluated herein (**a**) printing time (s), (**b**) part weight (g), (**c**) flexural strength (MPa), (**d**) EPC (MJ).

To identify which parameter values have a strong influence on each metric studied, box plots were formed for four critical metrics in the work (Figure 6). This is identified by the distribution of the calculated experimental values of the metric for a specific value of the control parameter. Low distribution (values are gathered together) shows that this specific value of the control parameter has a weak influence on the specific metric. The high distribution of the calculated experimental values of the metric for a specific value of the control parameter indicates a strong influence on the metric. According to these criteria, the following are observed in Figure 6:

- Printing time (s): LT (mm) values show mostly high distribution. For the 0.25 mm and 0.30 mm LT values, printing time values are gathered around three values. This is not a clear compact response though. For the PS (mm/s) control parameter, printing time (s) values are scattered in all its levels, except 70 mm/s. In this case, again printing time values are gathered around three values.
- Part weight (g): All levels of the ID (%) and the PS (mm/s) control parameters result in a high distribution in the part weight (g) values.
- Flexural strength (MPa): For the RDA (deg) in all levels, the flexural strength (MPa) values are gathered around two values, except for the 0 (deg) value, in which high distribution of the metric is observed. All levels of the NT (°C) control parameter result in high distribution of the flexural strength (MPa) values.
- EPC (MJ): Both LT (mm) and ID (%) control parameters in all their levels resulted in a high distribution of the EPC (MJ) values.

The data in the boxplots suggest that more research is needed to comprehend how the 3D printing settings affect the mechanical performance in the flexural test and the energy consumption metrics.

**Figure 7:**
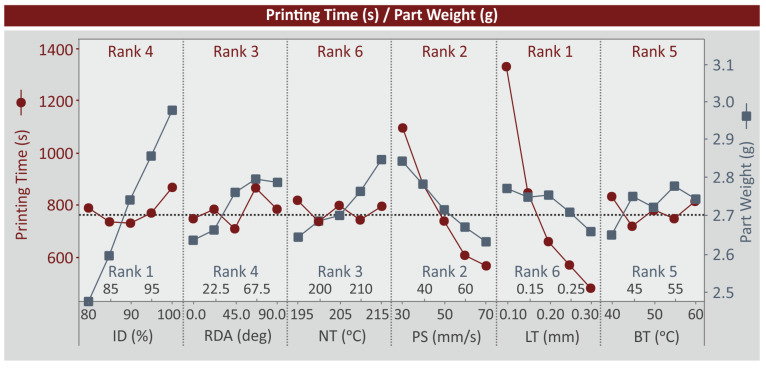
MEP: printing time (s), part weight (g).

**Figure 8:**
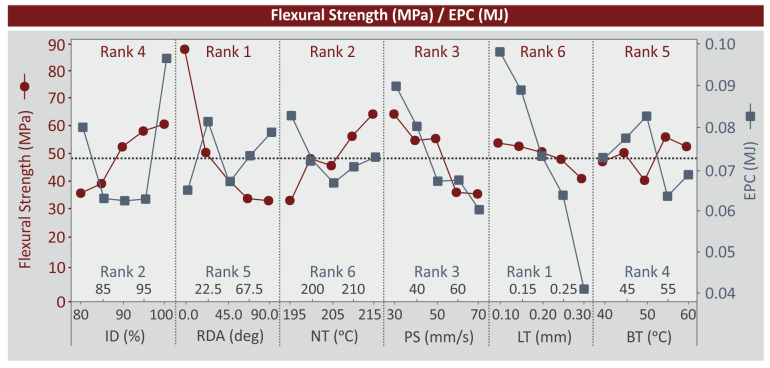
MEP: flexural strength (MPa), EPC (MJ).

MEPs indicate the effect of each control parameter level on a specific metric studied. Figure 7 and Figure 8 show the MEP for four of the metrics studied, while MEPs for the remaining metrics are available in the Supplementary Data for the study. In the MEP, the control parameters are ranked according to their importance in the metric. For the printing time (s) (Figure 7), ID (%), RDA (deg), NT (°C), and BT (°C) do not significantly affect the metric. LT (mm) and PS (mm/s) increase and reduce the printing time (s) and are ranked as no.1 and no.2 control parameters for the metric. For the part weight (g), RDA (reg), LT (mm), and BT (°C) do not significantly affect the metric. ID (%) is ranked as the number 1 control parameter and its increase increases the part weight (g). The same effect was found with the increase of the NT (°C). The increase of PS (mm/s) decreases the part weight (g) and is ranked as rank 2. Both the printing time (s) and the part weight (g) are related to the energy metrics studied (please see Equations (4) and (5)).

For the flexural strength (MPa) (Figure 8), LT (mm) and BT (°C) have a moderate influence on the metric. The increase of ID (%) and NT (°C) increases flexural strength (MPa). On the other hand, the increase of RDA (deg) and PS (mm/s) have the opposite effect (flexural strength (MPa) decreases). RDA (deg) is the dominant parameter (rank no.1) and LT (mm) is the least important one (rank no.6). For the EPC (MJ) metric, the increase of LT (mm) and PS (mm/s) decreases the metric. RDA (deg) and BT (°C) have a zig-zag response. The RDA (deg) 0 and 45 deg and the BT (°C) 55 °C minimize the EPC (MJ) metric. ID (%) median values reduce the EPC (MJ) while the lowest and highest value increase the EPC (MJ). The mid value of NT (°C) minimized the EPC (MJ), which is higher in higher and lower values of NT (°C). LT (mm) is the dominant parameter for the EPC (MJ) and NT (°C) is the least important one.

**Figure 9:**
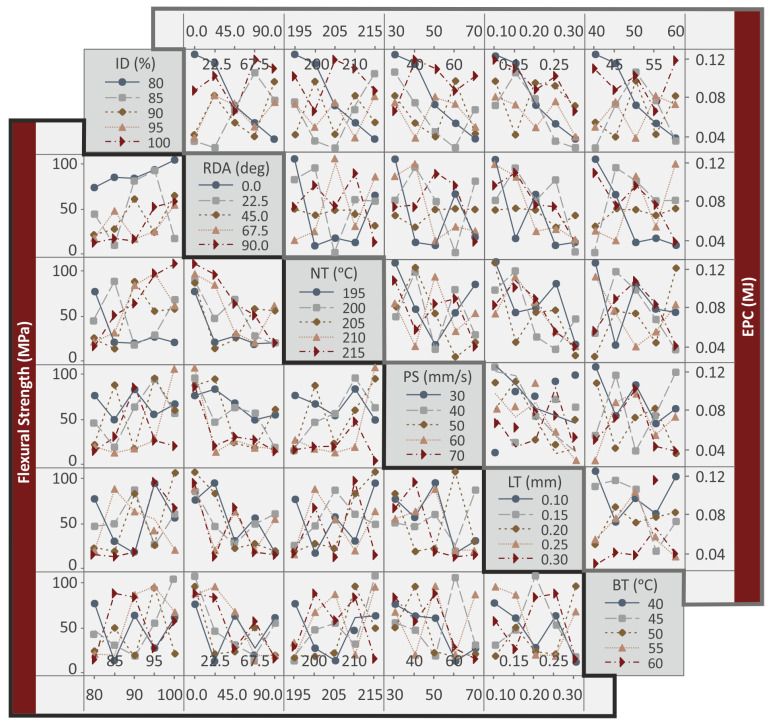
Interaction plots: flexural strength (MPa), EPC (MJ).

Although the MEPs show the effect of each control parameter in each response metric studied, it does not provide any information about the relations between the control parameters in each metric. To determine such interactions, interaction plots are required that show the synergistic and antagonistic relations between the control parameters. They were formed in the work for all the eight metrics (response indicators) studied and are shown in Figure 9 and the Supplementary Data for the work. Figure 9 shows the interaction plots for the six control parameters for the flexural strength (MPa) and the EPC (MJ) metrics studied. In all cases, antagonistic relations are observed between the control parameters.

### 3.4. Regression Analysis

The Reduced Quadratic Regression Model (RQRM) for each response is calculated:

(6) $$Y_{k}=a_{k}+\overset{n}{\underset{i=1}{\sum }}b_{i,k}x_{i}+\overset{n}{\underset{i=1}{\sum }}c_{i,k}x_{i}^{2}+e_{k}$$

where k represents the quality output (e.g., weight, printing time, flexural strength, flexural modulus of elasticity, flexural toughness, EPC, SPE, SPP), a is the constant value, b is the coefficient of the linear terms, c is the coefficient of the quadratic terms, e is the error, and x_i_ the seven (n = 6) control parameters, i.e., the Infill Density, Raster Deposition Angle, Nozzle Temperature, Printing Speed, Layer Thickness, and Bed Temperature.

From the ANOVA for each metric, a table was formed and a corresponding prediction model as a function of the six control parameters. For the metrics studied, these analysis results are presented in Table 4, Table 5, Table 6 and Table 7 and Equations (7)–(10). For the remaining metrics studied, the corresponding data are available in the Supplementary Data supplied.

In all the metrics, the *p*-value was close to zero and F-value was high (much higher than 4) indicating that the models sufficiently fit the data and the null hypothesis can be rejected. Regression values higher than 93.98% were found for the printing time (s), 92.88% for the part weight (g), 89.61% for the flexural strength (MPa), and 82.25% for the EPC (MJ). For the remaining metrics, regression values are close to 80%, and only for the SPP (kW/g) metric regression values are about 71%, which is still an acceptable value. These regression values indicate the sufficiency of the produced prediction models from the regression analysis conducted.

(7) $$\begin{array}{ll} \mathrm{PrintingTime}= & 30193-170.2\times \mathrm{ID}+0.815\times \mathrm{RDA}-167.8\times \mathrm{NT}\\ & -39.48\times \mathrm{PS}-14127\times \mathrm{LT}-73.0\times \mathrm{BT}+0.967\times {\mathrm{ID}}^{2}\\ & -0.00106\times {\mathrm{RDA}}^{2}+0.407\times {\mathrm{NT}}^{2}+0.2618\times {\mathrm{PS}}^{2}\\ & +25407\times {\mathrm{LT}}^{2}+0.727\times {\mathrm{BT}}^{2} \end{array}$$

(8) $$\begin{array}{ll} \mathrm{Weight}=11.30 & +0.0431\times \mathrm{ID}+0.003652\times \mathrm{RDA}-0.1353\times \mathrm{NT}\\ & -0.01003\times \mathrm{PS}+0.667\times \mathrm{LT}+0.0572\times \mathrm{BT}\\ & -0.000101\times {\mathrm{ID}}^{2}-0.000019\times {\mathrm{RDA}}^{2}+0.000353\times {\mathrm{NT}}^{2}\\ & +0.000048\times {\mathrm{PS}}^{2}-2.99\times {\mathrm{LT}}^{2}-0.000528\times {\mathrm{BT}}^{2} \end{array}$$

(9) $$\begin{array}{ll} \mathrm{sB}=-963+6.38 & \times \mathrm{ID}-1.5098\times \mathrm{RDA}+6.38\times \mathrm{NT}-0.594\times \mathrm{PS}\\ & +79.4\times \mathrm{LT}-3.09\times \mathrm{BT}-0.0278\times {\mathrm{ID}}^{2}\\ & +0.010551\times {\mathrm{RDA}}^{2}-0.0120\times {\mathrm{NT}}^{2}-0.00172\times {\mathrm{PS}}^{2}\\ & -349\times {\mathrm{LT}}^{2}+0.0342\times {\mathrm{BT}}^{2} \end{array}$$

(10) $$\begin{array}{ll} \mathrm{EPC}=6.58- & 0.05190\times \mathrm{ID}+0.000096\times \mathrm{RDA}-0.0411\times \mathrm{NT}\\ & -0.002075\times \mathrm{PS}-0.058\times \mathrm{LT}+0.00611\times \mathrm{BT}\\ & +0.000292\times {\mathrm{ID}}^{2}-0.000000\times {\mathrm{RDA}}^{2}+0.000099\times {\mathrm{NT}}^{2}\\ & +0.000013\times {\mathrm{PS}}^{2}-0.548\times {\mathrm{LT}}^{2}-0.000065\times {\mathrm{BT}}^{2} \end{array}$$

**Figure 10:**
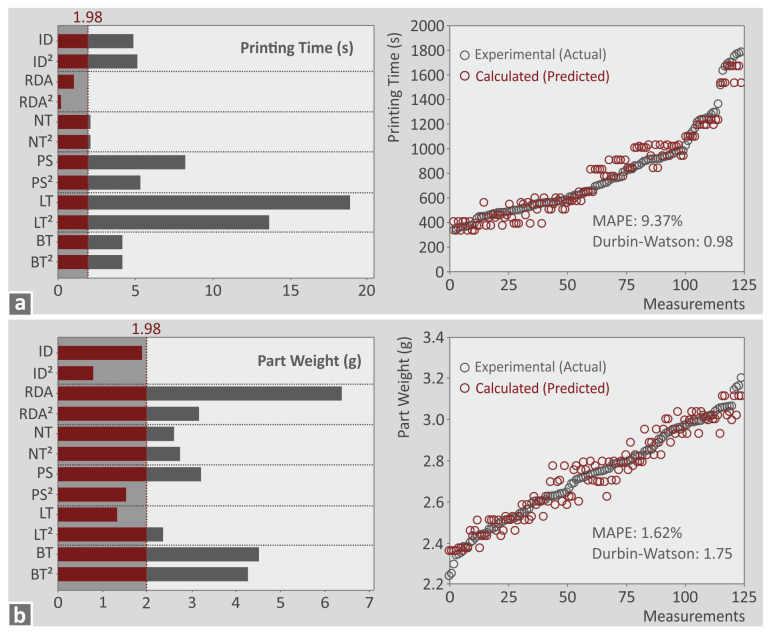
Pareto diagram along with a graph depicting a comparison between the experimental and the calculated values of the following metrics: (**a**) printing time (s), (**b**) part weight (g).

**Figure 11:**
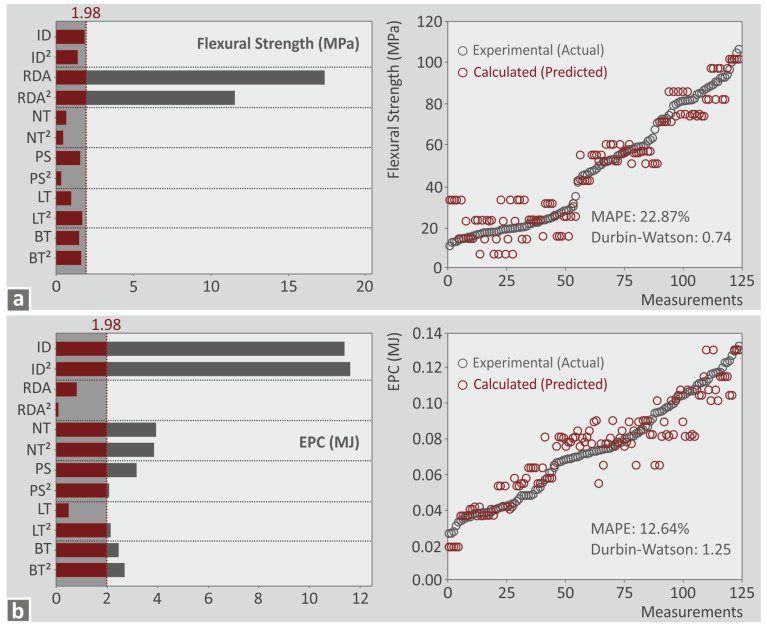
Pareto diagram along with a graph depicting a comparison between the experimental and the calculated values of the following metrics: (**a**) flexural strength (MPa), (**b**) EPC (MJ).

Further in the analysis, to identify the statistically important parameters, Pareto charts were formed for each metric (Figure 10 and Figure 11 and in the Supplementary Data provided). Parameters crossing the 1.98 margin in the Pareto chart are considered statistically important for the specific metric. With the prediction models formed, the metrics values were calculated and compared to the corresponding experimental values. Next to the Pareto chart for each metric, a graph with a comparison between the experimental and the calculated values are provided. Two indicators were calculated to evaluate the deviation between the values, i.e., the Mean Absolute Percentage Error (MAPE) and the Durbin–Watson indicator. The MAPE indicator is an estimation of the prediction accuracy. Acceptable values are those lower than 5 % [65]. The Durbin–Watson indicator shows the autocorrelation of the corresponding prediction model. Values less than 2 indicate a positive autocorrelation of the prediction model, equal to 2 show no autocorrelation, and higher than 2 indicate negative autocorrelation [66]. According to the above, the following are observed:

- Printing time (s): all parameters except RDA and RDA^2^ are statistically important (Figure 10a). MAPE was higher than 5% but lower than 10%, which is considered an acceptable accuracy [67]. Durbin–Watson of 0.98 shows a positive autocorrelation.
- Part weight (g): all parameters except ID, ID^2^, PS^2^, and LT are statistically important (Figure 10b). MAPE was 1.62% which is a very good result. Durbin–Watson of 1.75 shows a negative autocorrelation.
- Flexural strength (MPa): RDA and RDA^2^ were the only statistically important parameters (Figure 11a). MAPE was 22.87% which indicated that the accuracy of the prediction model is reduced, but still within reasonable limits (less than 25%) [68]. Durbin–Watson of 0.74 shows a positive autocorrelation.
- EPC (MJ): all parameters except RDA, RDA^2^, and LT are statistically important (Figure 11b). MAPE was 12.64% which is a good forecast [68]. Durbin–Watson of 1.25 shows a negative autocorrelation.

**Figure 12:**
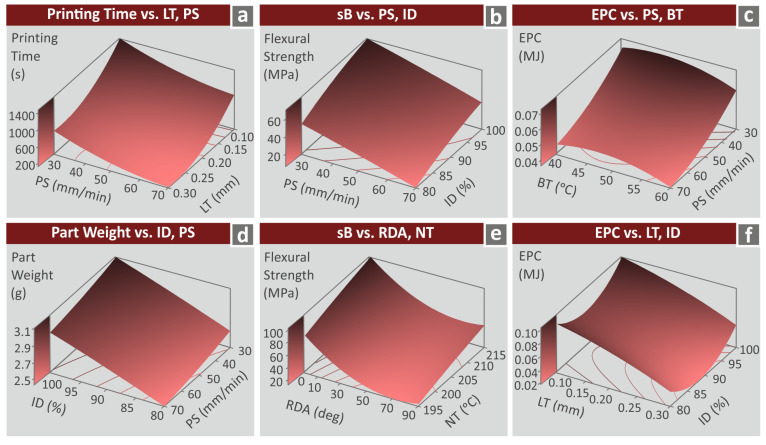
Surface 3D diagram showing the effect of two input parameters with high rank for the following metrics (**a**) printing time (s) vs. LT, PS, (**b**) flexural strength (MPa) vs. PS, ID, (**c**) EPC (MJ) vs. PS, BT, (**d**) part weight (g) vs. PS, ID, (**e**) sb (MPa) vs. RDA, NT, (**f**) EPC (MJ) vs. LT, ID.

The effect of two (high-ranked) combined control parameters for each metric is presented in the following Figure 12 in a three-dimensional surface graph. Four metrics are depicted, i.e., printing time (s), part weight (g), flexural strength (MPa), and EPC (MJ). PS and LT were the control parameters ranked as no.1 and no.2 for the printing time. Figure 12a shows the printing time vs. these two control parameters. Figure 12b shows the sB metric vs. PS and ID control parameters (rank no.3 and no.4, respectively). Figure 12c shows the EPC control metric vs. PS and BT (rank no.3 and no.4, respectively). Figure 12d shows the part weight vs. the ID and the PS which were also ranked as no.1 and no.2 control parameters for the specific metric. Figure 12e shows the sB metric vs. RDA and NT control parameters (rank no.1 and no.2, respectively). Finally, Figure 12f shows the EPC control metric vs. LT and ID (rank no.1 and no.2, respectively).

### 3.5. Confirmation Runs

To further evaluate the accuracy of the prediction models, two additional experimental runs (run 26 and 27) were conducted with five replicas each. Their control parameter values are shown in Table 8. These values were selected according to the optimum values that maximize the flexural strength (MPa) in run 26 and minimize the EPC (MJ) in run 27, as these two (flexural strength and EPC) are the most critical metrics for the study. The experimental results (mean values and deviation) for the metrics studied are shown in Table 9 and Table 10. More analytically the experimental results are presented in the Supplementary Data, in which corresponding tables are provided with the values of the metrics for each experimental repetition.

Table 11 compares the experimental results in the two confirmation runs with the corresponding calculated values with the prediction models for the two most critical metrics in the study, i.e., flexural strength (MPa) and EPC (MJ). The error between the corresponding values is calculated and presented in Table 11. For the flexural strength (MPa), the error for both runs is less than twenty percent (19.28% and 15.44% respectively for run 26 and run 27), which is acceptable deviation. For the EPC (MJ) metric, the accuracy for run 26 was even better, with a 13.11 % calculated deviation. In run 27 the model failed to predict the EPC (MJ), so no reliable value for the error was possible to calculate. The failure of the calculation of the specific metric is owed to the fact that the set of values of the 3D printing settings was probably outside the range of the specific prediction model in which the EPC (MJ) metric operates. This shows that the modeling process has limitations and operates in a specific range of values, which needs to be determined. Such an investigation was not within the scope of the current study.

## 4. Discussion

PLA is the most popular material in FFF MEX 3D printing, thoroughly studied in the literature, as presented above. Nevertheless, the energy consumed during the manufacturing of parts with the PLA polymer has not been reported in the literature so far. This gap is covered by the current research, which additionally investigates how six generic machine-independent parameters of the FFF MEX 3D printing process affect the energy requirements. It was found that, in a similar way to the mechanical properties, the 3D printing settings highly affect energy consumption. Differences in the experimental findings were high when altering the values of the 3D printing settings for both the energy consumption and the flexural properties. Modeling tools were employed for the analysis of these effects, to identify their relations and interactions and locate a possible set of parameters that optimize these contradicted parts’ performance aspects, i.e., minimize energy consumption, which contributes to the sustainability of the process and maximize the mechanical properties in the flexural test. Overall, it was found that a balance between the energy demands when 3D printing, and PLA parts and their mechanical properties can be achieved. This means that median, in their mechanical properties, parts can be produced using moderate energy amounts. For specific 3D printing settings, the effect is contradictory. For example, 70 mm/s PS reduces energy consumption, but it has the same effect on mechanical properties. The same applies for the 90 deg RDA value. A 100% ID produced parts with high flexural strength but required high energy consumption for their construction; 0 deg RDA produced parts with high flexural strength, with consumption reduced for the specific control parameter energy, but this amount was average compared to the other control parameters and levels.

Regarding the flexural properties of the FFF MEX 3D-printed PLA parts, the effect of the 3D printing settings on the performance of the parts has been thoroughly investigated. Studies have investigated similar 3D printing settings and values to the current study and in most cases, the results are in good agreement [69,70,71]. Still, there are studies reporting values for flexural strength higher than the ones presented herein [72] and others that present lower values [73]. For the analysis of the experimental findings, a similar approach to the current study is often used (statistical analysis and modeling) [72]. Such an approach was necessary, due to the number of the parameters studied and by considering the obtained experimental results, which vary for the flexural strength for example from about 35 MPa to almost 90 MPa.

It should be noted that in the three-point flexural stress measurements described by the applied ASTM D790-02, the tensile and compressive stress vectors are parallel to the 3D printed layers during the deformation (stress within layers). Since the interlayer adhesion is a crucial element when assessing the mechanical properties of FFF 3D printed parts, printing the parts in the vertical direction would assess how the 3D printing parameters can affect the interlayer adhesion, with the aim of minimizing the strong loss of mechanical properties along the interlayer direction. Still, 3D printing the samples vertically requires significantly more time; therefore, it is not usually selected as a 3D printing strategy. Additionally, the increase in the 3D printing time would negatively affect the energy consumption for the parts, as it was found that 3D printing time highly affects the consumed energy.

Although the sustainability of FFF MEX 3D printing has been investigated for various aspects, for example, the determination of the required energy for the recycling of the PLA material in a form suitable for the process [74], the consumed energy during the process itself has not been reported yet in the literature. Therefore, no experimental data can be compared with the findings presented herein. Such a study has been presented for the ABS polymer [34], reporting that the energy demands for the fabrication of parts with the ABS polymer with the FFF MEX 3D printing process are reduced by increasing the PS and the LT 3D printing settings. As mentioned, these findings refer to a different polymer (ABS), with the study investigating the effect of the 3D printing settings also on the tensile properties of the ABS parts built with the FFF 3D printing process. Still, these findings regarding the dominant parameters affecting energy consumption are in very good agreement with the findings presented herein. In both studies, the increase of PS and LT reduced the energy demands for the 3D printing of the parts with the FFF process.

From the analysis, a strong correlation between the EPC and the printing time was found. LT and PS were rank no.1 and no.2 parameters for the printing time. The increase in the values of these two parameters reduces the printing time, which is the same effect as the EPC metric. EPC, as mentioned, also has LT and PS ranked as the no.1 and no.3 control parameters, respectively. It is rather impressive that the parts built with the lowest LT value of 0.10 mm need to be built with about nine times the energy of the parts built with the highest LT value (0.30 mm) require. ID is ranked as the no.2 control parameter for the EPC metric. Low and high values increase the EPC, while the median values reduce the EPC metric. An increase of 240% in the energy demands is reported between the parts built with the highest ID value and median ID values. RDA levels result in a zig-zag EPC response, with the lowest RDA value (0 deg) having low energy demands, then the EPC metric increased (22.5 deg), then it decreased again (45 deg), and it constantly increased up to the highest RDA value tested. Surprisingly, the two temperature control parameters (NT and BT) are ranked as no.4 and no.6, showing a rather small influence of the temperature in the energy demands during MEX 3D printing.

## 5. Conclusions

Herein, the energy for the production of parts with the PLA polymer with the FFF MEX 3D printing process was quantified, providing tangible information for the evaluation of the sustainability of the process. Six generic machine-independent parameters with three levels each were studied to determine how they affect the energy consumption of the FFF MEX 3D printing process. A strong influence was found since the difference in the energy demands reached 250% among the cases studied. This by itself justifies the need for such an analysis. At the same time, the effect of these six 3D printing settings on the flexural strength of the PLA parts made with the FFF MEX 3D printing process was also evaluated. Again, a strong effect of the 3D printing settings on the flexural strength of the produced PLA parts was found, with differences reaching 300% among the cases studied. An effort was made to identify a set of 3D printing setting values that produce parts with optimized performance (minimum energy requirements and high flexural strength). This was not possible to achieve, although specific parameters, such as the 0 deg RDA (the dominant parameter for the flexural strength), minimize the energy consumption and maximize the flexural strength. Minimum energy consumption was achieved with high LT (the dominant parameter for energy consumption) values, which at the same time produce parts with average flexural strength. Thus, according to the priorities and the specifications of each application, suitable parameters can be used. The prediction models derived from the regression analysis proved their accuracy with two additional experimental runs. Therefore, they can be used for the estimation of the metrics of interest in each application.

The confirmation runs revealed the limitations of the prediction models, with the energy consumption model failing to predict values in the second confirmation run. This showed that the produced models operate within limits that need to be determined. All the indicators of the statistical analysis showed that the models were more than sufficient to estimate the corresponding metrics studied. Only in the case of the flexural strength the MAPE value indicated that a reasonable deviation is expected in the prediction for the metric. In future work, the boundaries of the models provided can be identified and extended by widening the control parameters list and their values. Additionally, different 3D printing orientations can be investigated, as explained in the discussion section.

## Figures and Tables

**Table 1:** Taguchi L25 Design: Control parameters and levels.

Run	ID	RDA	NT	PS	LT	BT
1	80	0.0	195	30	0.10	40
2	80	22.5	200	40	0.15	45
3	80	45.0	205	50	0.20	50
4	80	67.5	210	60	0.25	55
5	80	90.0	215	70	0.30	60
6	85	0.0	200	50	0.25	60
7	85	22.5	205	60	0.30	40
8	85	45.0	210	70	0.10	45
9	85	67.5	215	30	0.15	50
10	85	90.0	195	40	0.20	55
11	90	0.0	205	70	0.15	55
12	90	22.5	210	30	0.20	60
13	90	45.0	215	40	0.25	40
14	90	67.5	195	50	0.30	45
15	90	90.0	200	60	0.10	50
16	95	0.0	210	40	0.30	50
17	95	22.5	215	50	0.10	55
18	95	45.0	195	60	0.15	60
19	95	67.5	200	70	0.20	40
20	95	90.0	205	30	0.25	45
21	100	0.0	215	60	0.20	45
22	100	22.5	195	70	0.25	50
23	100	45.0	200	30	0.30	55
24	100	67.5	205	40	0.10	60
25	100	90.0	210	50	0.15	40

**Table 2:** Average and standard deviations values of measured responses for weight, flexural strength (sB), flexural modulus of elasticity (E), and flexural toughness.

Run	Weight (g)	sB (MPa)	E (MPa)	Toughness (MJ/m^3^)
1	2.38 ± 0.04	74.09 ± 1.59	2636.98 ± 37.86	2.36 ± 0.24
2	2.47 ± 0.05	45.85 ± 1.87	1848.62 ± 57.23	1.73 ± 0.08
3	2.50 ± 0.05	22.93 ± 0.63	1201.63 ± 47.36	0.97 ± 0.20
4	2.56 ± 0.04	19.63 ± 1.39	1152.99 ± 33.56	0.70 ± 0.12
5	2.51 ± 0.04	15.56 ± 0.80	1013.51 ± 17.51	0.39 ± 0.11
6	2.45 ± 0.03	84.74 ± 2.80	2506.45 ± 5.24	1.91 ± 0.15
7	2.30 ± 0.06	13.40 ± 1.26	1025.90 ± 90.87	0.44 ± 0.10
8	2.64 ± 0.04	30.00 ± 3.51	1426.69 ± 108.63	0.96 ± 0.22
9	2.91 ± 0.05	48.27 ± 2.64	1845.97 ± 42.01	1.44 ± 0.07
10	2.70 ± 0.07	19.26 ± 2.80	1043.26 ± 77.10	0.57 ± 0.09
11	2.60 ± 0.04	83.60 ± 1.62	2711.29 ± 74.98	2.54 ± 0.25
12	2.87 ± 0.05	80.56 ± 2.98	2519.13 ± 45.74	2.72 ± 0.15
13	2.85 ± 0.04	61.16 ± 2.04	2344.94 ± 38.64	2.20 ± 0.04
14	2.67 ± 0.06	18.82 ± 1.11	1511.72 ± 98.13	0.39 ± 0.09
15	2.74 ± 0.05	17.59 ± 0.69	1519.80 ± 59.37	0.62 ± 0.14
16	2.78 ± 0.03	92.27 ± 2.23	2693.10 ± 64.00	3.03 ± 0.05
17	2.99 ± 0.05	91.60 ± 2.60	2900.55 ± 26.48	3.14 ± 0.13
18	2.78 ± 0.05	26.10 ± 2.68	2121.53 ± 53.40	0.88 ± 0.26
19	2.73 ± 0.06	27.04 ± 1.81	2126.07 ± 78.79	0.62 ± 0.09
20	3.01 ± 0.04	53.24 ± 1.84	2663.80 ± 67.32	0.54 ± 0.08
21	2.99 ± 0.04	103.32 ± 3.90	3100.88 ± 61.20	3.39 ± 0.13
22	2.71 ± 0.07	20.25 ± 0.60	1960.63 ± 183.12	0.62 ± 0.10
23	3.06 ± 0.06	64.95 ± 5.26	2783.28 ± 96.50	0.99 ± 0.15
24	3.13 ± 0.06	55.08 ± 2.45	2918.48 ± 103.46	0.80 ± 0.21
25	3.00 ± 0.04	58.68 ± 1.46	2740.21 ± 17.01	0.67 ± 0.14

**Table 3:** Average and standard deviations values of measured responses for printing time, EPC, SPE, and SPP.

Run	Printing Time (s)	EPC (MJ)	SPE (MJ/g)	SPP (kW/g)
1	1721.80 ± 41.70	0.124 ± 0.009	0.052 ± 0.005	0.030 ± 0.003
2	862.20 ± 19.85	0.115 ± 0.010	0.047 ± 0.004	0.054 ± 0.004
3	586.60 ± 22.96	0.071 ± 0.004	0.029 ± 0.002	0.049 ± 0.004
4	431.60 ± 18.85	0.053 ± 0.008	0.021 ± 0.003	0.048 ± 0.007
5	334.40 ± 13.78	0.037 ± 0.001	0.015 ± 0.000	0.044 ± 0.003
6	447.80 ± 18.07	0.035 ± 0.002	0.014 ± 0.001	0.032 ± 0.003
7	346.20 ± 17.02	0.028 ± 0.003	0.012 ± 0.001	0.035 ± 0.004
8	916.40 ± 29.36	0.071 ± 0.006	0.027 ± 0.002	0.030 ± 0.003
9	1233.40 ± 30.42	0.105 ± 0.010	0.036 ± 0.004	0.029 ± 0.004
10	733.60 ± 35.70	0.076 ± 0.006	0.028 ± 0.003	0.039 ± 0.004
11	550.20 ± 29.49	0.042 ± 0.002	0.016 ± 0.001	0.029 ± 0.003
12	935.00 ± 31.04	0.081 ± 0.014	0.028 ± 0.005	0.030 ± 0.006
13	624.00 ± 16.81	0.054 ± 0.011	0.019 ± 0.004	0.030 ± 0.007
14	464.20 ± 20.19	0.039 ± 0.003	0.015 ± 0.001	0.032 ± 0.002
15	1086.60 ± 57.81	0.096 ± 0.007	0.035 ± 0.003	0.032 ± 0.002
16	510.20 ± 29.37	0.038 ± 0.003	0.014 ± 0.001	0.027 ± 0.003
17	1273.80 ± 48.97	0.081 ± 0.007	0.027 ± 0.003	0.021 ± 0.002
18	672.20 ± 22.43	0.072 ± 0.005	0.026 ± 0.002	0.039 ± 0.003
19	545.20 ± 12.56	0.049 ± 0.003	0.018 ± 0.001	0.033 ± 0.002
20	846.60 ± 48.38	0.074 ± 0.004	0.025 ± 0.001	0.029 ± 0.002
21	502.00 ± 52.07	0.087 ± 0.013	0.029 ± 0.004	0.058 ± 0.007
22	494.00 ± 25.74	0.102 ± 0.006	0.038 ± 0.002	0.076 ± 0.004
23	750.20 ± 28.40	0.065 ± 0.006	0.021 ± 0.002	0.029 ± 0.003
24	1663.80 ± 107.66	0.119 ± 0.005	0.038 ± 0.002	0.023 ± 0.001
25	922.20 ± 44.35	0.110 ± 0.005	0.037 ± 0.002	0.040 ± 0.003

**Table 4:** Polynomial ANOVA, printing time vs. ID, RDA, NT, PS, LT, and BT.

Source	DF	AdjSS	AdjMS	F-Value	*p*-Value
Regression	12	16,414,303	1,367,859	184.11	0.000
ID	1	195,386	195,386	26.30	0.000
RDA	1	6761	6761	0.91	0.342
NT	1	36,635	36,635	4.93	0.028
PS	1	538,015	538,015	72.42	0.000
LT	1	2,670,110	2,670,110	359.39	0.000
BT	1	116,130	116,130	15.63	0.000
ID^2^	1	204,539	204,539	27.53	0.000
RDA^2^	1	100	100	0.01	0.908
NT^2^	1	36,271	36,271	4.88	0.029
PS^2^	1	239,940	239,940	32.30	0.000
LT^2^	1	1,412,050	1,412,050	190.06	0.000
BT^2^	1	115,716	115,716	15.57	0.000
Error	112	832,114	7430		
Total	124	17,246,417	1,375,289		
R^2^	95.18%				
R^2^ (adj)	94.66%				
R^2^ (pred)	93.98%				

**Table 5:** Polynomial ANOVA, weight vs. ID, RDA, NT, PS, LT, and BT.

Source	DF	AdjSS	AdjMS	F-Value	*p*-Value
Regression	12	6.05821	0.504851	154.07	0.000
ID	1	0.01254	0.012538	3.83	0.053
RDA	1	0.13582	0.135821	41.45	0.000
NT	1	0.02382	0.023820	7.27	0.008
PS	1	0.03472	0.034723	10.60	0.001
LT	1	0.00594	0.005944	1.81	0.181
BT	1	0.07126	0.071259	21.75	0.000
ID^2^	1	0.00224	0.002243	0.68	0.410
RDA^2^	1	0.03239	0.032391	9.89	0.002
NT^2^	1	0.02732	0.027316	8.34	0.005
PS^2^	1	0.00797	0.007968	2.43	0.122
LT^2^	1	0.01958	0.019583	5.98	0.016
BT^2^	1	0.06104	0.061037	18.63	0.000
Error	112	0.36699	0.003277		
Total	124	6.42520	0.508128		
R^2^	94.29%				
R^2^ (adj)	93.68%				
R^2^ (pred)	92.88%				

**Table 6:** Polynomial ANOVA, sB vs. ID, RDA, NT, PS, LT, and BT.

Source	DF	AdjSS	AdjMS	F-Value	*p*-Value
Regression	12	93,044.6	7753.7	101.81	0.000
ID	1	274.7	274.7	3.61	0.060
RDA	1	23,211.8	23,211.8	304.79	0.000
NT	1	52.9	52.9	0.69	0.406
PS	1	121.6	121.6	1.60	0.209
LT	1	84.2	84.2	1.11	0.295
BT	1	208.3	208.3	2.73	0.101
ID^2^	1	169.5	169.5	2.23	0.139
RDA^2^	1	9985.9	9985.9	131.12	0.000
NT^2^	1	31.5	31.5	0.41	0.521
PS^2^	1	10.4	10.4	0.14	0.713
LT^2^	1	265.8	265.8	3.49	0.064
BT^2^	1	256.4	256.4	3.37	0.069
Error	112	8529.5	76.2		
Total	124	101,574.1	7829.9		
R^2^	91.60%				
R^2^ (adj)	90.70%				
R^2^ (pred)	89.61%				

**Table 7:** Polynomial ANOVA, EPC vs. ID, RDA, NT, PS, LT, and BT.

Source	DF	AdjSS	AdjMS	F-Value	*p*-Value
Regression	12	0.090255	0.007521	56.10	0.000
ID	1	0.018164	0.018164	135.47	0.000
RDA	1	0.000093	0.000093	0.70	0.406
NT	1	0.002196	0.002196	16.38	0.000
PS	1	0.001486	0.001486	11.08	0.001
LT	1	0.000045	0.000045	0.34	0.562
BT	1	0.000813	0.000813	6.07	0.015
ID^2^	1	0.018645	0.018645	139.06	0.000
RDA^2^	1	0.000001	0.000001	0.01	0.935
NT^2^	1	0.002151	0.002151	16.04	0.000
PS^2^	1	0.000636	0.000636	4.75	0.031
LT^2^	1	0.000656	0.000656	4.89	0.029
BT^2^	1	0.000937	0.000937	6.99	0.009
Error	112	0.015017	0.000134		
Total	124	0.105272	0.007655		
R^2^	85.74%				
R^2^ (adj)	84.21%				
R^2^ (pred)	82.25%				

**Table 8:** Control parameters for the confirmation runs.

Run	ID	RDA	NT	PS	LT	BT
26	100	0.0	215	30	0.11	60
27	88.9	0.0	207.1	70	0.30	60

**Table 9:** Average and standard deviations values of measured responses for weight, flexural strength, flexural modulus of elasticity, and flexural toughness, for the confirmation runs.

Run	Weight (g)	sB (MPa)	E (MPa)	Toughness (MJ/m^3^)
26	3.60 ± 0.08	114.75 ± 5.19	4826.61 ± 107.65	4.46 ± 0.08
27	2.09 ± 0.07	61.69 ± 1.19	1893.41 ± 69.59	1.75 ± 0.02

**Table 10:** Average and standard deviations values of measured responses for printing time, EPC, SPE, and SPP, for the confirmation runs.

Run	Printing Time (s)	EPC (MJ)	SPE (MJ/g)	SPP (kW/g)
26	1360.60 ± 31.79	0.136 ± 0.010	0.038 ± 0.003	0.028 ± 0.002
27	274.20 ± 38.32	0.022 ± 0.001	0.010 ± 0.001	0.038 ± 0.006

**Table 11:** Validation table.

Run		26	27
Actual	sB (MPa)	114.75	61.69
	EPC (MJ)	0.14	0.02
Predicted	sB (MPa)	136.87	71.22
	EPC (MJ)	0.12	N/A
Absolute Error	sB (%)	19.28	15.44
	EPC (%)	13.11	N/A

## Data Availability

The data presented in this study are available upon request from the corresponding author.

## Supplementary Materials

The following supporting information can be downloaded at https://www.mdpi.com/article/10.3390/polym15051232/s1, Figure S1. MEP: flexural modulus of elasticity (MPa), tensile toughness (MJ/m3); Figure S2. MEP: SPE (MJ/g), SPP (kW/g); Figure S3. Interaction plots: printing time (s), part weight (g); Figure S4. Interaction plots: flexural modulus of elasticity (MPa), tensile toughness (MJ/m3); Figure S5. Interaction plots: SPE (MJ/g), SPP (kW/g); Figure S6. Pareto and experimental vs. calculated charts: (a) flexural modulus of elasticity (MPa), (b) tensile toughness (MJ/m3); Figure S7. Pareto and experimental vs. calculated charts: (a) SPE (MJ/g), (b) SPP (kW/g); Table S1. Measured Weight, Flexural Strength, Flexural Modulus of Elasticity, and Flexural Toughness for each experimental run and five replicas per run; Table S2. Measured for Printing Time, EPC, SPE, SPP for each experimental run and five replicas per run; Table S3. Polynomial ANOVA, E vs. ID, RDA, NT, PS, LT, BT; Table S4. Polynomial ANOVA, Toughness vs. ID, RDA, NT, PS, LT, BT; Table S5. Polynomial ANOVA, SPE vs. ID, RDA, NT, PS, LT, BT; Table S6. Polynomial ANOVA, SPP vs. ID, RDA, NT, PS, LT, BT; Table S7. Measured Weight, Flexural Strength, Flexural Modulus of Elasticity, and Flexural Toughness for each experimental run and five replicas per run for the Confirmation Runs; Table S8. Measured Printing Time, EPC, SPE, SPP for each experimental run and five replicas per run for the Confirmation Runs.

## Nomenclature

3DP	3D Printing
ABS	Acrylonitrile Butadiene Styrene
AM	Additive Manufacturing
ANOVA	Analysis of Variances
BT	Bed Temperature
DF	Degrees of Freedom
DOE	Design of Experiment
DSC	Differential Scanning Calorimetry
E	Tensile Modulus of Elasticity
EPC	Energy Printing Consumption
FFF	Fused Filament Fabrication
ID	Infill Density
LT	Layer Thickness
MEP	Main Effect Plot
MEX	Material Extrusion
NT	Nozzle Temperature
ORA	Orientation Angle
PA	Polyamide
PC	Polycarbonate
PMMA	Poly(methyl methacrylate)
PLA	Polylactic Acid
PT	Printing Time
PS	Printing Speed
RDA	Raster Deposition Angle
QRM	Quadratic Regression Model
RQRM	Reduced Quadratic Regression Model
sB	Compression Strength
SEM	Scanning Electron Microscopy
SPE	Specific Printing Energy
SPP	Specific Printing Power
Tg	Glass Transition Temperature
TGA	Thermogravimetric Analysis
