# Supplementary material for: Functionality Versus Sustainability for PLA in MEX 3D Printing: The Impact of Generic Process Control Factors on Flexural Response and Energy Efficiency
Source: Polymers (Basel). 2023 Feb 28;15(5):1232. doi: 10.3390/polym15051232 (PMC10007265; doi:10.3390/polym15051232)
Supplement: Supplementary file 1 [file polymers-15-01232-s001.zip › polymers-2228220-supplementary.pdf]

polymers

MDPI

Supplementary material for the article

Functionality versus sustainability for PLA in MEX 3D Printing: The impact of generic process control factors on flexural response and energy efficiency

Markos Petousis [1], Nectarios Vidakis [1*], Nikolaos Mountakis [1], Emmanuel Karapidakis [3], Amalia Moutsopoulou [1]

[1] Department of Mechanical Engineering, Hellenic Mediterranean University, Heraklion 71410, Greece
[2] Electrical and Computer Engineering Dept., Hellenic Mediterranean University, Heraklion 71410, Greece
* Correspondence: e-mail: vidakis@hmu.gr (Prof. Nectarios Vidakis, N.V.), Tel.: +302810379227

Abstract: Process sustainability vs mechanical strength is a strong market-driven claim in Material Extrusion (MEX) Additive Manufacturing (AM). Especially for the most popular polymer, Polylactic Acid (PLA), the concurrent achievement of these opposing goals may become a puzzle, especially since MEX 3D-printing offers a variety of process parameters. Herein, multi-objective optimization of material deployment, 3D printing flexural response, and energy consumption in MEX AM with PLA is introduced. To evaluate the impact of the most important generic and device-independent control parameters on these responses, the Robust Design theory was employed. Raster Deposition Angle (RDA), Layer Thickness (LT), Infill Density (ID), Nozzle Temperature (NT), Bed Temperature (BT), and Printing Speed (PS) were selected to compile a five-level orthogonal array. Twenty-five experimental runs with five specimen replicas each, accumulated 135 experiments in total. Analysis of variances and reduced quadratic regression models (RQRM) were used to decompose the impact of each parameter on the responses. The LT, ID, RDA, and LT were ranked first in impact on printing time, material weight, flexural strength, and energy consumption respectively. The RQRM predictive models were experimentally validated and hold significant technological merit, for the proper adjustment of process control parameters per the MEX 3D-printing case.

Keywords: Polylactic Acid (PLA); Optimization; Material Extrusion (MEX); Energy consumption; Energy efficiency, Flexural Strength; Taguchi Analysis; Robust Design

3. Results

3.2. DOE and experimental results

Table S1. Measured Weight, Flexural Strength, Flexural Modulus of Elasticity, and Flexural Toughness for each experimental run and five replicas per run.

A/A	Run	Weight (g)	sB (MPa)	E (MPa)	Toughness (MJ/m³)
1		2.383	76.67	2614.11	2.63
2		2.347	73.66	2607.11	2.56
3	1	2.441	72.34	2701.29	2.22
4		2.354	73.71	2623.59	2.06
5		2.383	74.07	2638.80	2.32
6		2.479	47.67	1911.78	1.87
7	2	2.506	46.50	1890.28	1.75
8		2.523	42.70	1764.44	1.68
9		2.404	45.93	1832.02	1.71

10		2.425	46.46	1844.58	1.67
11		2.433	22.94	1184.51	1.15
12		2.494	23.07	1152.46	1.03
13	3	2.550	23.91	1279.63	0.76
14		2.469	22.35	1203.20	1.17
15		2.530	22.39	1188.37	0.75
16		2.594	21.19	1160.70	0.55
17		2.607	20.72	1101.34	0.82
18	4	2.513	18.06	1194.65	0.60
19		2.544	18.36	1149.66	0.78
20		2.522	19.82	1158.59	0.75
21		2.468	15.95	1005.58	0.25
22		2.473	16.44	1041.44	0.56
23	5	2.516	14.36	994.97	0.35
24		2.567	15.18	1008.40	0.41
25		2.518	15.85	1017.17	0.40
26		2.443	80.90	2499.20	1.75
27		2.451	82.88	2513.33	1.75
28	6	2.502	86.38	2506.03	2.02
29		2.411	87.83	2509.05	2.09
30		2.446	85.69	2504.66	1.93
31		2.253	11.52	876.14	0.28
32		2.383	13.71	1121.10	0.52
33	7	2.298	15.04	1039.83	0.44
34		2.241	13.24	1062.35	0.51
35		2.342	13.51	1030.05	0.47
36		2.628	28.68	1261.99	1.21
37		2.605	35.83	1520.34	0.61
38	8	2.606	26.52	1524.55	0.99
39		2.642	28.85	1439.43	1.00
40		2.713	30.10	1387.14	0.98
41		2.962	44.11	1912.92	1.52
42		2.949	48.05	1812.13	1.45
43	9	2.917	51.33	1812.16	1.45
44		2.885	49.34	1858.35	1.47
45		2.845	48.52	1834.28	1.33
46		2.628	19.55	951.83	0.48
47		2.749	15.37	989.95	0.56
48	10	2.632	23.02	1152.09	0.65
49		2.748	18.18	1054.66	0.48
50		2.754	20.16	1067.80	0.68
51		2.563	82.32	2639.06	2.27
52	11	2.605	82.55	2666.35	2.61
53		2.546	86.33	2833.67	2.92

54		2.648	83.07	2697.30	2.39
55		2.631	83.73	2720.09	2.50
56		2.852	75.53	2469.74	2.59
57		2.944	81.69	2589.32	2.95
58	12	2.855	82.51	2504.48	2.80
59		2.802	80.27	2535.68	2.66
60		2.904	82.82	2496.43	2.60
61		2.820	62.93	2286.01	2.16
62		2.791	60.52	2379.48	2.18
63	13	2.872	60.23	2367.69	2.22
64		2.851	63.53	2325.90	2.19
65		2.906	58.58	2365.61	2.26
66		2.668	20.10	1350.06	0.35
67		2.689	17.29	1607.52	0.36
68	14	2.603	19.65	1567.31	0.26
69		2.760	18.72	1511.17	0.47
70		2.612	18.33	1522.55	0.49
71		2.724	17.13	1457.71	0.52
72		2.789	18.32	1611.80	0.85
73	15	2.799	16.65	1495.98	0.63
74		2.717	18.07	1492.30	0.59
75		2.692	17.81	1541.19	0.51
76		2.735	93.71	2642.65	3.03
77		2.784	89.91	2798.40	3.11
78	16	2.824	91.91	2647.25	3.02
79		2.789	90.54	2671.17	2.99
80		2.750	95.29	2706.01	2.99
81		3.013	94.13	2932.69	3.30
82		3.021	89.31	2862.26	2.98
83	17	2.937	94.07	2903.20	3.04
84		3.040	91.90	2914.38	3.19
85		2.932	88.58	2890.22	3.20
86		2.791	24.98	2136.57	0.74
87		2.828	24.29	2034.59	0.67
88	18	2.719	30.83	2176.48	1.25
89		2.823	24.87	2145.15	1.07
90		2.762	25.54	2114.84	0.69
91		2.730	24.17	2008.30	0.74
92		2.787	28.99	2221.33	0.58
93	19	2.736	27.39	2109.10	0.66
94		2.640	26.70	2166.05	0.64
95		2.776	27.94	2125.56	0.49
96	20	2.988	50.79	2662.94	0.48
97		3.015	52.38	2567.52	0.48

98		2.985	54.11	2749.06	0.67
99		2.973	53.25	2642.17	0.49
100		3.065	55.70	2697.29	0.56
101		2.993	107.91	3150.67	3.55
102		2.954	101.70	3138.46	3.44
103	21	2.974	97.95	2996.55	3.20
104		2.963	106.14	3114.84	3.32
105		3.056	102.89	3103.90	3.44
106		2.811	19.98	2177.65	0.59
107		2.629	20.53	1924.47	0.69
108	22	2.742	19.33	1679.64	0.69
109		2.643	20.54	2040.35	0.68
110		2.708	20.85	1981.04	0.46
111		3.049	68.58	2719.26	1.22
112		3.158	71.78	2940.10	1.06
113	23	3.017	59.01	2695.48	0.84
114		3.010	64.34	2760.35	0.89
115		3.062	61.06	2801.23	0.93
116		3.144	58.79	3043.91	0.65
117		3.058	56.27	2760.91	0.95
118	24	3.164	53.78	2962.36	0.50
119		3.203	53.84	2901.21	1.00
120		3.060	52.72	2924.03	0.91
121		2.960	59.01	2723.79	0.57
122		2.991	57.84	2762.98	0.90
123	25	3.064	59.84	2722.89	0.56
124		2.987	56.59	2747.89	0.67
125		2.995	60.10	2743.49	0.63
	Min:	2.241	11.52	876.14	0.25
	Max:	3.203	107.91	3150.67	3.55
	Average:	2.732	49.12	2092.70	1.39

Table S2. Measured for Printing Time, EPC, SPE, SPP for each experimental run and five replicas per run.

A/A	Run	Printing Time (s)	EPC (MJ)	SPE (MJ/g)	SPP (kW/g)
1		1696	0.116	0.049	0.029
2		1696	0.132	0.056	0.033
3	1	1763	0.112	0.046	0.026
4		1771	0.130	0.055	0.031
5		1683	0.129	0.054	0.032
6		842	0.103	0.042	0.049
7		842	0.111	0.044	0.053
8	2	864	0.126	0.050	0.058
9		884	0.123	0.051	0.058
10		879	0.111	0.046	0.052
11		590	0.077	0.032	0.053
12		585	0.074	0.030	0.051
13	3	609	0.068	0.027	0.044
14		549	0.070	0.028	0.052
15		600	0.067	0.026	0.044
16		427	0.047	0.018	0.042
17		408	0.060	0.023	0.056
18	4	424	0.042	0.017	0.040
19		441	0.057	0.022	0.050
20		458	0.060	0.024	0.052
21		339	0.035	0.014	0.041
22		312	0.037	0.015	0.048
23	5	349	0.037	0.015	0.043
24		333	0.039	0.015	0.045
25		339	0.037	0.015	0.043
26		425	0.037	0.015	0.036
27		450	0.033	0.014	0.030
28	6	470	0.032	0.013	0.028
29		459	0.037	0.015	0.033
30		435	0.034	0.014	0.032
31		354	0.025	0.011	0.032
32		325	0.030	0.012	0.038
33	7	369	0.026	0.011	0.031
34		348	0.032	0.014	0.041
35		335	0.025	0.011	0.032
36		899	0.066	0.025	0.028
37		923	0.079	0.030	0.033
38	8	901	0.076	0.029	0.032
39		894	0.067	0.025	0.029
40		965	0.069	0.026	0.027
41	9	1219	0.098	0.033	0.027

42		1236	0.102	0.035	0.028
43		1227	0.117	0.040	0.033
44		1283	0.094	0.033	0.025
45		1202	0.116	0.041	0.034
46		703	0.081	0.031	0.044
47		740	0.069	0.025	0.034
48	10	732	0.077	0.029	0.040
49		790	0.084	0.031	0.039
50		703	0.071	0.026	0.037
51		598	0.041	0.016	0.027
52		553	0.040	0.015	0.027
53	11	519	0.045	0.018	0.034
54		544	0.040	0.015	0.028
55		537	0.044	0.017	0.031
56		966	0.094	0.033	0.034
57		966	0.063	0.021	0.022
58	12	897	0.094	0.033	0.037
59		913	0.082	0.029	0.032
60		933	0.073	0.025	0.027
61		614	0.047	0.017	0.027
62		600	0.073	0.026	0.043
63	13	631	0.047	0.016	0.026
64		642	0.050	0.018	0.028
65		633	0.051	0.018	0.028
66		447	0.041	0.015	0.034
67		457	0.037	0.014	0.030
68	14	456	0.041	0.016	0.035
69		499	0.042	0.015	0.031
70		462	0.036	0.014	0.030
71		1009	0.097	0.036	0.035
72		1152	0.106	0.038	0.033
73	15	1100	0.089	0.032	0.029
74		1048	0.090	0.033	0.032
75		1124	0.099	0.037	0.033
76		516	0.037	0.014	0.027
77		493	0.041	0.015	0.030
78	16	532	0.035	0.012	0.023
79		541	0.036	0.013	0.024
80		469	0.039	0.014	0.030
81		1352	0.086	0.029	0.021
82		1225	0.075	0.025	0.020
83	17	1243	0.084	0.029	0.023
84		1283	0.072	0.024	0.018
85		1266	0.086	0.029	0.023

86		690	0.079	0.028	0.041
87		672	0.068	0.024	0.036
88	18	634	0.071	0.026	0.041
89		679	0.076	0.027	0.040
90		686	0.068	0.025	0.036
91		561	0.047	0.017	0.031
92		540	0.047	0.017	0.031
93	19	540	0.054	0.020	0.037
94		555	0.048	0.018	0.032
95		530	0.050	0.018	0.034
96		900	0.081	0.027	0.030
97		899	0.072	0.024	0.027
98	20	815	0.073	0.025	0.030
99		812	0.073	0.025	0.030
100		807	0.073	0.024	0.029
101		426	0.072	0.024	0.056
102		500	0.080	0.027	0.054
103	21	561	0.104	0.035	0.062
104		485	0.096	0.032	0.067
105		538	0.082	0.027	0.050
106		504	0.104	0.037	0.074
107		476	0.097	0.037	0.078
108	22	478	0.095	0.035	0.072
109		535	0.106	0.040	0.075
110		477	0.107	0.040	0.083
111		741	0.056	0.018	0.025
112		797	0.071	0.022	0.028
113	23	720	0.069	0.023	0.032
114		749	0.066	0.022	0.029
115		744	0.066	0.022	0.029
116		1627	0.120	0.038	0.023
117		1661	0.122	0.040	0.024
118	24	1780	0.122	0.039	0.022
119		1506	0.112	0.035	0.023
120		1745	0.117	0.038	0.022
121		944	0.103	0.035	0.037
122		915	0.105	0.035	0.039
123	25	849	0.113	0.037	0.043
124		940	0.117	0.039	0.042
125		963	0.109	0.037	0.038
	Min:	312	0.025	0.011	0.018
	Max:	1780	0.132	0.056	0.083
	Average:	778.17	0.073	0.027	0.037

3.3. Statistical analysis

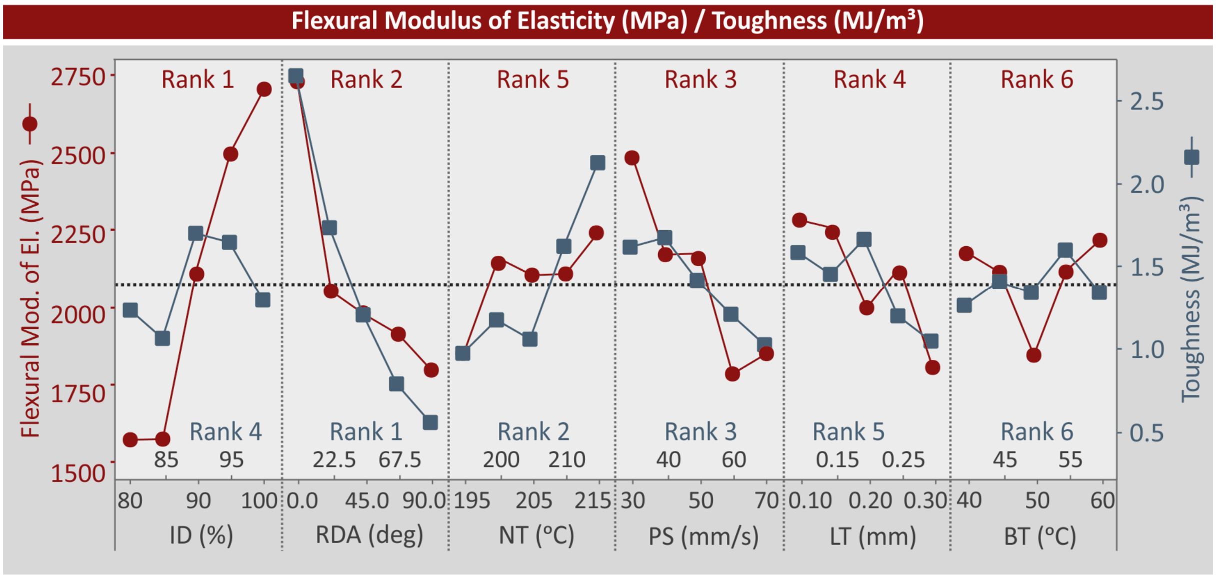

Figure S1. MEP: flexural modulus of elasticity (MPa), tensile toughness (MJ/m³)

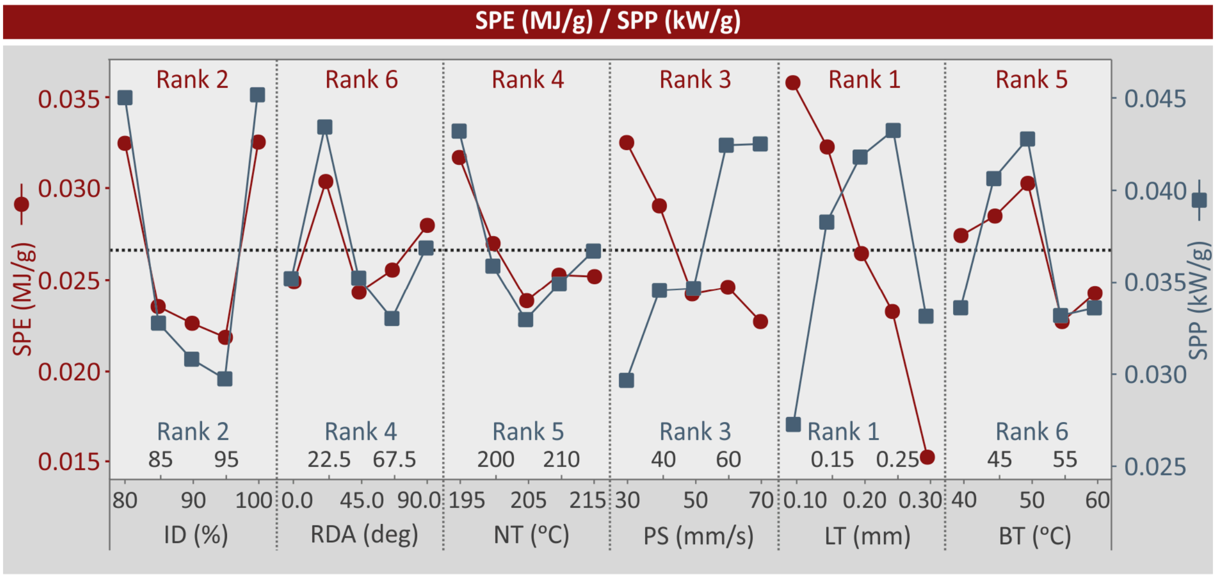

Figure S2. MEP: SPE (MJ/g), SPP (kW/g)

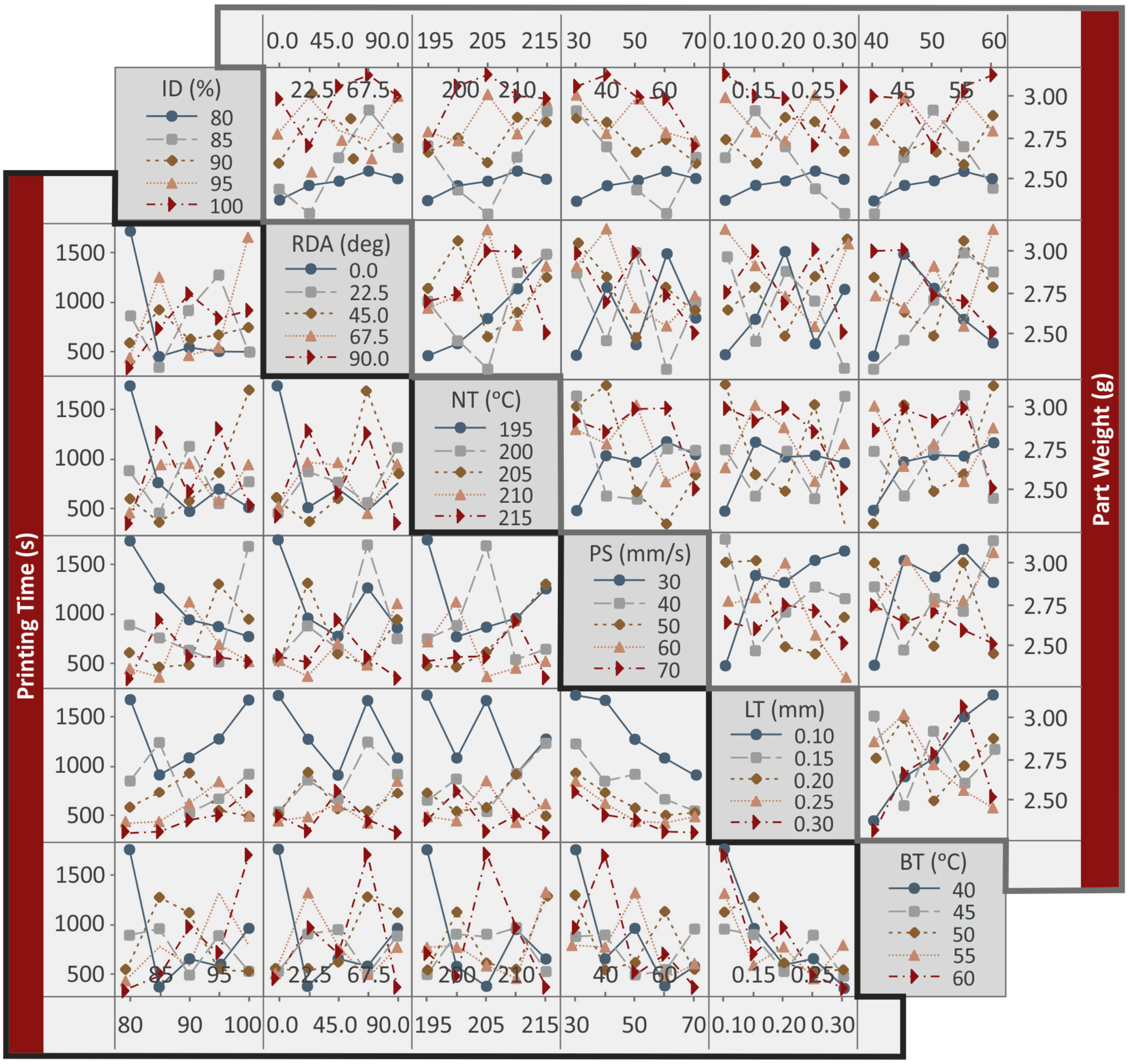

Figure S3. Interaction plots: printing time (s), part weight (g)

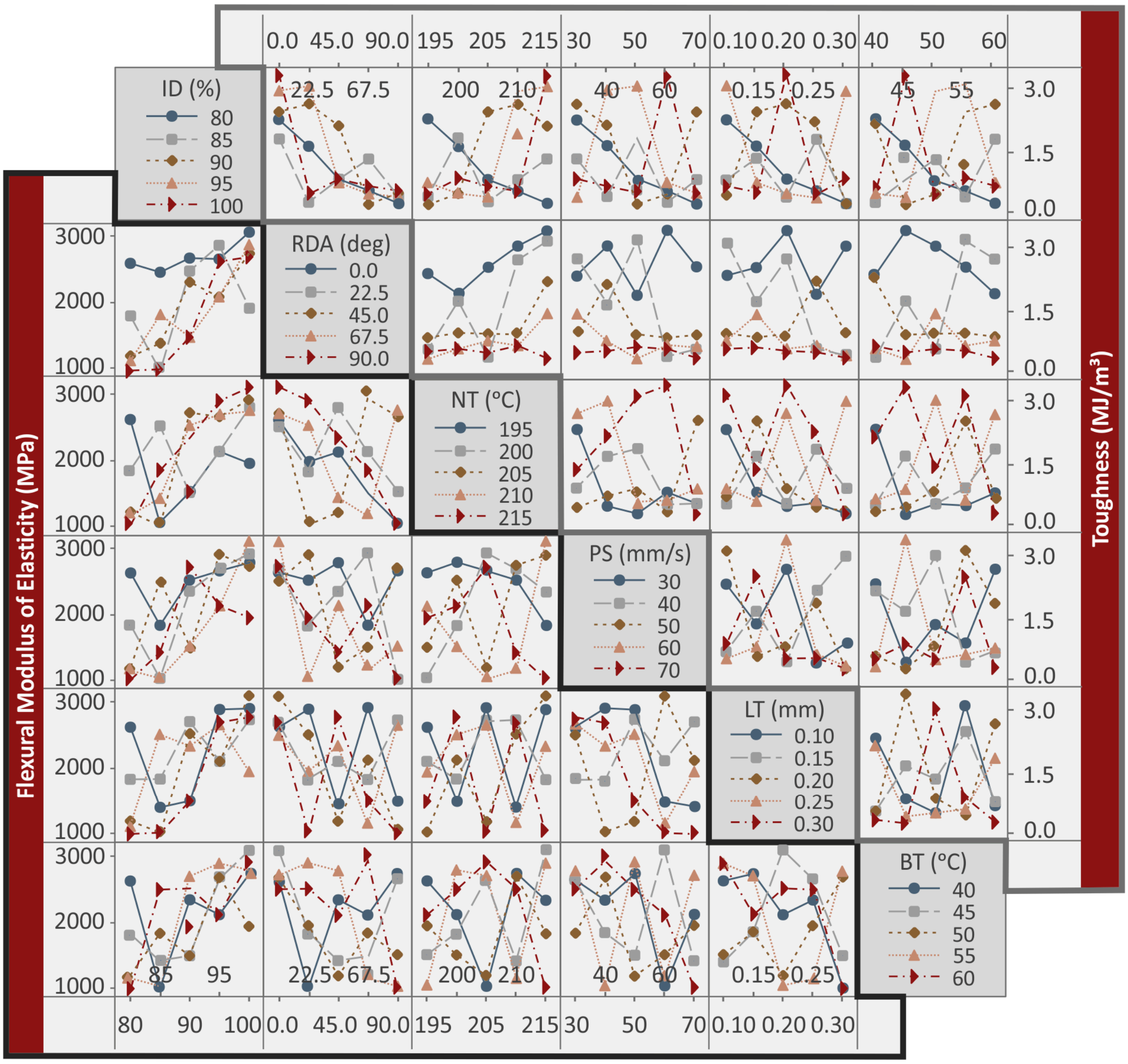

Figure S4. Interaction plots: flexural modulus of elasticity (MPa), tensile toughness (MJ/m³)

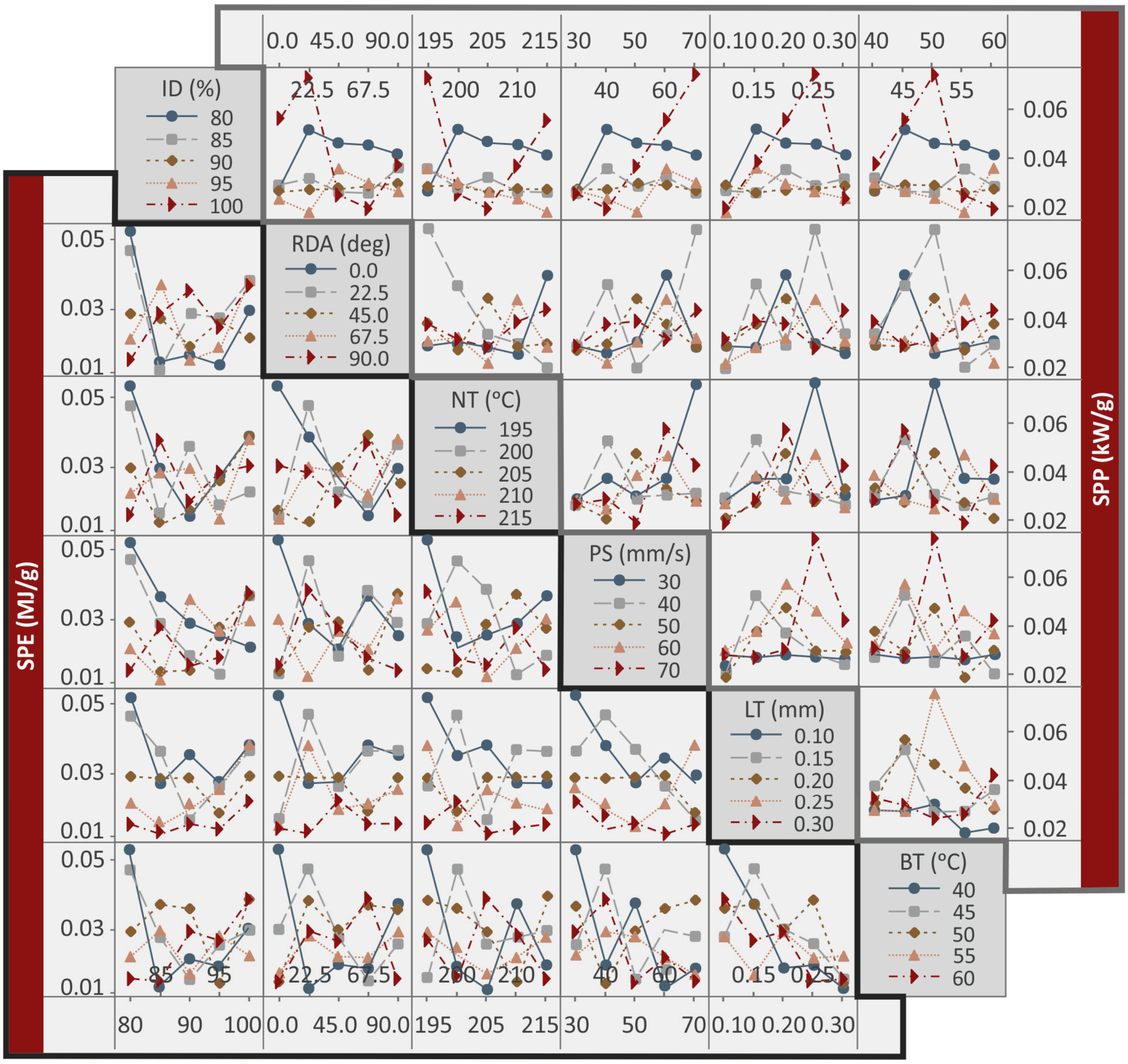

Figure S5. Interaction plots: SPE (MJ/g), SPP (kW/g)

3.4. Regression analysis

Table S3. Polynomial ANOVA, E vs ID, RDA, NT, PS, LT, BT.

Source	DF	AdjSS	AdjMS	F-Value	P-Value
Regression	12	50941002	4245083	74.35	0.000
ID	1	19806	19806	0.35	0.557
RDA	1	5565239	5565239	97.47	0.000
NT	1	152275	152275	2.67	0.105
PS	1	656560	656560	11.50	0.001
LT	1	397	397	0.01	0.934
BT	1	1309728	1309728	22.94	0.000
ID^2	1	94035	94035	1.65	0.202
RDA^2	1	2314511	2314511	40.54	0.000
NT^2	1	139716	139716	2.45	0.121
PS^2	1	253549	253549	4.44	0.037

LT^2	1	74077	74077	1.30	0.257
BT^2	1	1333673	1333673	23.36	0.000
Error	112	6395015	57098		
Total	124	57336017	4302181		
R^2	88.85%				
R^2 (adj)	87.65%				
R^2 (pred)	86.29%				

$$E = -26676 - 54.2 \times ID - 23.38 \times RDA + 342 \times NT - 43.6 \times PS + 172 \times LT - 245.1 \times BT + 0.656 \times ID^2 + 0.1606 \times RDA^2 - 0.799 \times NT^2 + 0.269 \times PS^2 - 5819 \times LT^2 + 2.469 \times BT^2 \quad \text{(S1)}$$

Table S4. Polynomial ANOVA, Toughness vs ID, RDA, NT, PS, LT, BT.

Source	DF	AdjSS	AdjMS	F-Value	P-Value
Regression	12	106.893	8.9077	96.02	0.000
ID	1	2.037	2.0373	21.96	0.000
RDA	1	17.538	17.5381	189.06	0.000
NT	1	2.587	2.5875	27.89	0.000
PS	1	0.076	0.0760	0.82	0.367
LT	1	0.400	0.4001	4.31	0.040
BT	1	0.426	0.4258	4.59	0.034
ID^2	1	1.937	1.9365	20.88	0.000
RDA^2	1	3.903	3.9035	42.08	0.000
NT^2	1	2.793	2.7931	30.11	0.000
PS^2	1	0.338	0.3384	3.65	0.059
LT^2	1	0.898	0.8978	9.68	0.002
BT^2	1	0.385	0.3853	4.15	0.044
Error	112	10.390	0.0928		
Total	124	117.283	9.0005		
R^2	91.14%				
R^2 (adj)	90.19%				
R^2 (pred)	89.03%				

$$Toughness = 112.5 + 0.550 \times ID - 0.04150 \times RDA - 1.410 \times NT + 0.0148 \times PS + 5.47 \times LT + 0.1398 \times BT - 0.002975 \times ID^2 + 0.000209 \times RDA^2 + 0.003573 \times NT^2 - 0.000311 \times PS^2 - 20.26 \times LT^2 - 0.001327 \times BT^2 \quad \text{(S2)}$$

Table S5. Polynomial ANOVA, SPE vs ID, RDA, NT, PS, LT, BT.

Source	DF	AdjSS	AdjMS	F-Value	P-Value
Regression	12	0.012114	0.001010	52.88	0.000
ID	1	0.002791	0.002791	146.18	0.000
RDA	1	0.000001	0.000001	0.05	0.831
NT	1	0.000352	0.000352	18.43	0.000
PS	1	0.000247	0.000247	12.91	0.000
LT	1	0.000012	0.000012	0.61	0.438

BT	1	0.000097	0.000097	5.06	0.026
ID^2	1	0.002784	0.002784	145.85	0.000
RDA^2	1	0.000002	0.000002	0.11	0.738
NT^2	1	0.000339	0.000339	17.77	0.000
PS^2	1	0.000128	0.000128	6.68	0.011
LT^2	1	0.000068	0.000068	3.58	0.061
BT^2	1	0.000121	0.000121	6.33	0.013
Error	112	0.002138	0.000019		
Total	124	0.014252	0.001029		

$$\begin{aligned} SPE = 2.647 &- 0.02034 \times ID - 0.000009 \times RDA - 0.01645 \times NT - 0.000845 \times PS \\ &- 0.0294 \times LT + 0.002105 \times BT + 0.000113 \times ID^2 + 0.000000 \times RDA^2 \\ &+ 0.000039 \times NT^2 + 0.000006 \times PS^2 - 0.1769 \times LT^2 - 0.000023 \times BT^2 \end{aligned} \tag{S3}$$

Table S6. Polynomial ANOVA, SPP vs ID, RDA, NT, PS, LT, BT.

Source	DF	AdjSS	AdjMS	F-Value	P-Value
Regression	12	0.015419	0.001285	31.77	0.000
ID	1	0.005618	0.005618	138.91	0.000
RDA	1	0.000000	0.000000	0.00	0.946
NT	1	0.000938	0.000938	23.19	0.000
PS	1	0.000081	0.000081	1.99	0.161
LT	1	0.003852	0.003852	95.24	0.000
BT	1	0.001057	0.001057	26.14	0.000
ID^2	1	0.005601	0.005601	138.48	0.000
RDA^2	1	0.000014	0.000014	0.34	0.559
NT^2	1	0.000918	0.000918	22.71	0.000
PS^2	1	0.000008	0.000008	0.19	0.666
LT^2	1	0.003458	0.003458	85.49	0.000
BT^2	1	0.001107	0.001107	27.37	0.000
Error	112	0.004530	0.000040		
Total	124	0.019949	0.001325		
R^2	77.29%				
R^2 (adj)	74.86%				
R^2 (pred)	71.91%				

$$\begin{aligned} SPP = 3.871 &- 0.02886 \times ID + 0.000004 \times RDA - 0.02685 \times NT + 0.000483 \times PS \\ &+ 0.5366 \times LT + 0.00696 \times BT + 0.000160 \times ID^2 - 0.000000 \times RDA^2 \\ &+ 0.000065 \times NT^2 - 0.000001 \times PS^2 - 1.257 \times LT^2 - 0.000071 \times BT^2 \end{aligned} \tag{S4}$$

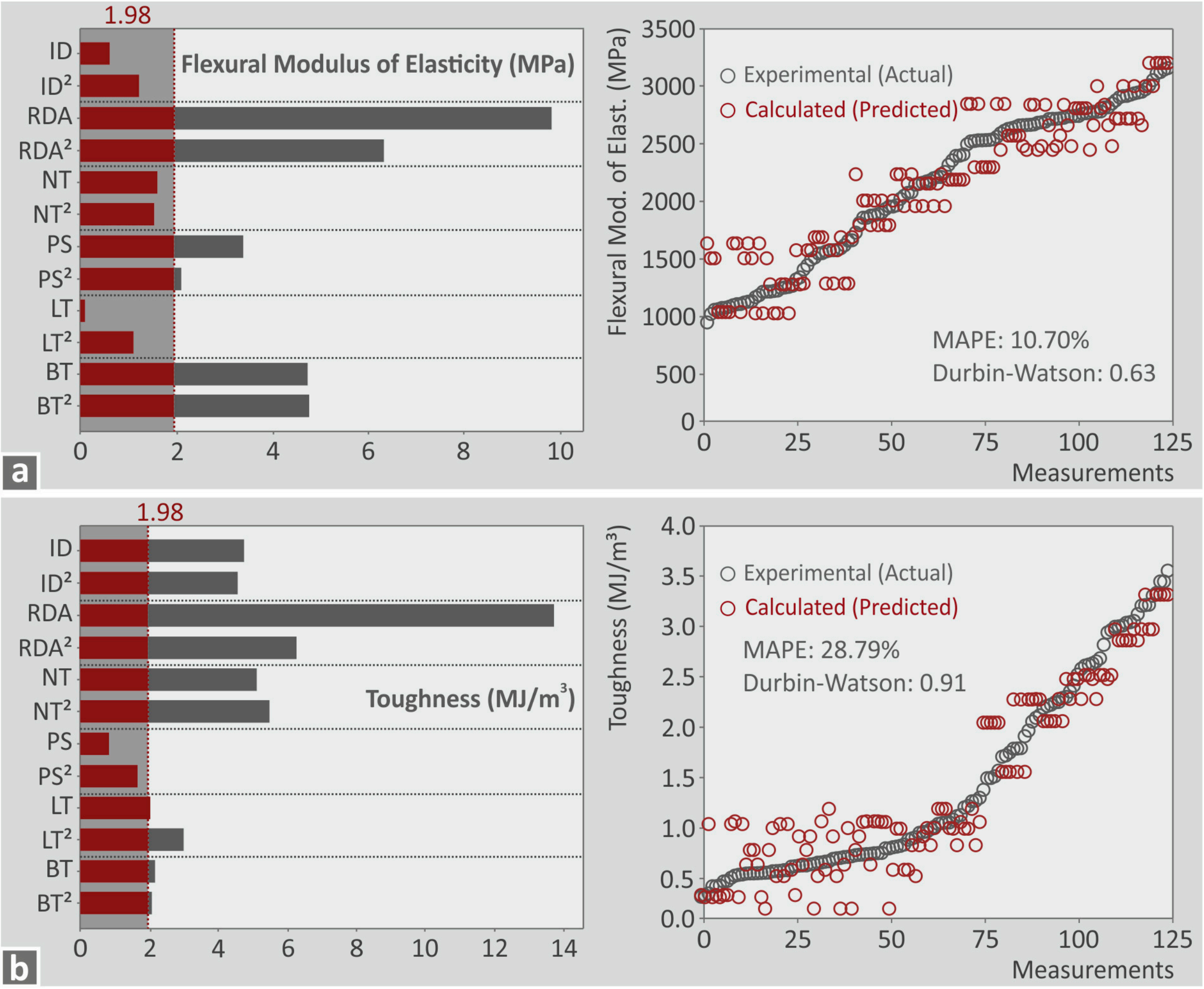

Figure S6. Pareto and experimental vs calculated charts: (a) flexural modulus of elasticity (MPa), (b) tensile toughness (MJ/m^3)

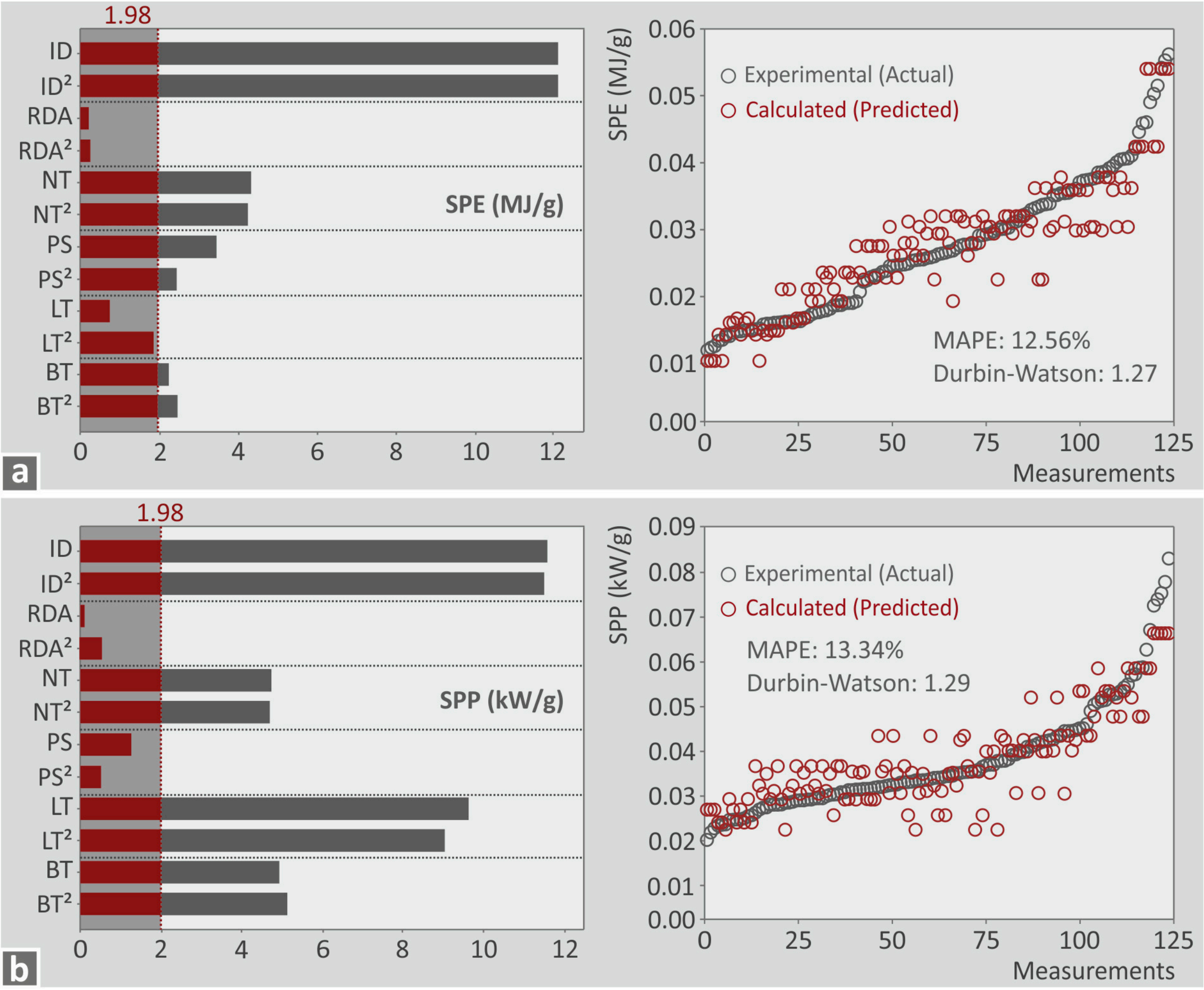

Figure S7. Pareto and experimental vs calculated charts: (a) SPE (MJ/g), (b) SPP (kW/g)

3.5. Confirmation run

Table S7. Measured Weight, Flexural Strength, Flexural Modulus of Elasticity, and Flexural Toughness for each experimental run and five replicas per run for the Confirmation Runs.

A/A	Run	Weight (g)	sB (MPa)	E (MPa)	Toughness (MJ/m³)
1		3.716	115.02	4923.50	4.47
2		3.621	116.01	4864.78	4.51
3	26	3.568	106.19	4693.79	4.55
4		3.503	116.26	4919.99	4.32
5		3.587	120.26	4730.99	4.46
6		1.979	62.28	1830.59	1.73
7		2.116	61.16	1816.70	1.78
8	27	2.178	61.77	1905.78	1.76
9		2.082	60.05	1931.44	1.76
10		2.102	63.23	1982.53	1.73
	Min:	1.979	60.05	1816.70	1.73
	Max:	3.716	120.26	4923.50	4.55
	Average:	2.845	88.22	3360.01	3.11

Table S8. Measured Printing Time, EPC, SPE, SPP for each experimental run and five replicas per run for the Confirmation Runs.

A/A	Run	Printing Time (s)	EPC (MJ)	SPE (MJ/g)	SPP (kW/g)
1		1318	0.143	0.038	0.029
2		1367	0.134	0.037	0.027
3	26	1405	0.120	0.034	0.024
4		1366	0.140	0.040	0.029
5		1347	0.143	0.040	0.029
6		246	0.022	0.011	0.046
7		287	0.021	0.010	0.035
8	27	224	0.021	0.010	0.044
9		318	0.023	0.011	0.035
10		296	0.021	0.010	0.033
	Min:	224	0.021	0.010	0.024
	Max:	1405	0.143	0.040	0.046
	Average:	817.40	0.079	0.024	0.033
